# Nano-Biotechnology in Soil Remediation: Use of Nanomaterials to Promote Plant Growth and Stress Tolerance

**DOI:** 10.3390/nano15221743

**Published:** 2025-11-19

**Authors:** Xunfeng Chen, Shuoqi Wang, Huijuan Lai, Linjing Deng, Qin Zhong, Charles Obinwanne Okoye, Qijian Niu, Yanping Jing, Juncai Wang, Jianxiong Jiang

**Affiliations:** 1 Key Laboratory of Zhenjiang, Biofuels Institute, School of Environment and Safety Engineering, School of Emergency Management, Collaborative Innovation Center of Technology and Material of Water Treatment, Jiangsu University, Zhenjiang 212013, China; xunfengchen@ujs.edu.cn (X.C.);; 2 Key Laboratory of Original Agro-Environmental Pollution Prevention and Control, Ministry of Agriculture and Rural Affairs (MARA)/Tianjin Key Laboratory of Agro-Environment and Agro-Product Safety, Agro-Environment Protection Institution, MARA, Tianjin 300191, China; 3 Dongzhu Ecological Environment Protection Co., Ltd., Wuxi 214101, China; 4 Department of Zoology & Environmental Biology, University of Nigeria, Nsukka 410001, Nigeria; 5 Guizhou Institute of Mountain Resources, Guizhou Academy of Sciences, Guiyang 550001, China

**Keywords:** nanobiotechnology, soil remediation, plant growth, heavy metal, nanomaterials

## Abstract

Soil degradation and pollution pose significant threats to global agricultural sustainability and food security. Conventional remediation methods are often constrained by low efficiency, high cost, and potential secondary pollution. Nanobiotechnology, an emerging interdisciplinary field, offers innovative solutions by integrating functional nanomaterials with plant–microbe interactions to advance soil remediation and sustainable agriculture. This review systematically elaborates on the mechanisms and applications of nanomaterials in soil remediation and enhanced plant stress resilience. For contaminant removal, nanomaterials such as nano-zero-valent iron (nZVI) and carbon nanotubes effectively immobilize or degrade heavy metals and organic pollutants through adsorption, catalysis, and other reactive mechanisms. In agriculture, nanofertilizers facilitate the regulated release of nutrients, thereby markedly enhancing nutrient use efficiency. Concurrently, certain nanoparticles mitigate a range of abiotic stresses—such as drought, salinity, and heavy metal toxicity—through the regulation of phytohormone balance, augmentation of photosynthetic performance, and reinforcement of antioxidant defenses. However, concerns regarding the environmental behavior, ecotoxicity, and long-term safety of nanomaterials remain. Future research should prioritize the development of smart, responsive nanosystems, elucidate the complex interactions among nanomaterials, plants, and microbes, and establish comprehensive life-cycle assessment and standardized risk evaluation frameworks. These efforts are essential to ensuring the safe and scalable application of nanobiotechnology in environmental remediation and green agriculture.

## 1. Introduction

Soil constitutes the fundamental substrate for agricultural productivity and global food security [1]. However, escalating anthropogenic activities have led to the accumulation of soil pollutants, resulting in severe land degradation worldwide [2]. According to the Food and Agriculture Organization (FAO), approximately 33% of global soils are currently degraded, primarily due to heavy metals, persistent organic pollutants (POPs), and microplastics [3].

Soil degradation encompasses various processes, including water erosion, desertification, salinization, nutrient depletion, and contamination [4]. Contemporary soil pollution has evolved beyond localized, single-contaminant scenarios, instead exhibiting characteristics of multimedia interactions, widespread composite pollution, and long-term latency [5]. Beyond traditional abiotic stressors, emerging contaminants such as antibiotic resistance genes (ARGs), per- and polyfluoroalkyl substances (PFAS), microplastics (MNPs), and nanoparticles (NPs) are increasingly being detected alongside conventional heavy metals like lead and cadmium [3,6,7].

A systematic framework of remediation has been established to address soil contamination [8]. Conventional methods are broadly categorized into physicochemical and biological approaches. Physicochemical techniques leverage the physicochemical properties of pollutants for separation, transformation, or immobilization. These include in situ technologies like chemical oxidation-reduction [9], electrokinetic remediation [10], and soil washing [11], as well as ex situ methods such as soil replacement [12], thermal desorption [13], and stabilization/solidification [14,15]. While mature, these techniques often suffer from limitations including narrow specificity, a focus on single contaminants, high costs, potential secondary pollution, and land use constraints [16]. Biological remediation, which utilizes plants, animals, and microorganisms to purify or stabilize pollutants, offers a less disruptive alternative [15]. However, it often requires extended timelines and struggles to address multi-medium composite pollution [16]. To overcome these drawbacks, combined remediation strategies have been explored. Notable approaches include microbial–animal–plant integration, chemical/physicochemical-biological coupling, and physical–chemical hybrid techniques [15]. These integrated methods aim to synergize the advantages of multiple technologies while mitigating individual limitations. For instance, Hrapovic et al. (2005) [17] elucidated the synergistic mechanism between potassium permanganate oxidation and bioremediation, demonstrating that manganese dioxide (MnO_2_) byproducts could inhibit microbial activity but also identifying adaptive mitigation pathways. Bai et al. (2019) [18] demonstrated that surfactant-enhanced soil washing coupled with electrochemical oxidation significantly improved phenanthrene removal while enabling washing solution regeneration. Guan et al. (2023) [19] reported that the plant growth-promoting rhizobacteria (PGPR) strain DLN5, capable of pyrene degradation, enhanced removal efficiency from 71.5% to 82.5% in a phytoremediation system, accelerating degradation kinetics by 15.4%. Advances in rhizosphere ecology have positioned plant–soil-microbe interactions as a pivotal focus for integrated phytoremediation. Cao et al. (2024) [20] showed that foliar application of zinc oxide nanoparticles (ZnO NPs) modulated root endophytic and rhizospheric microbial communities, alleviating phenanthrene-cadmium co-toxicity in lettuce.

**Figure 1:**
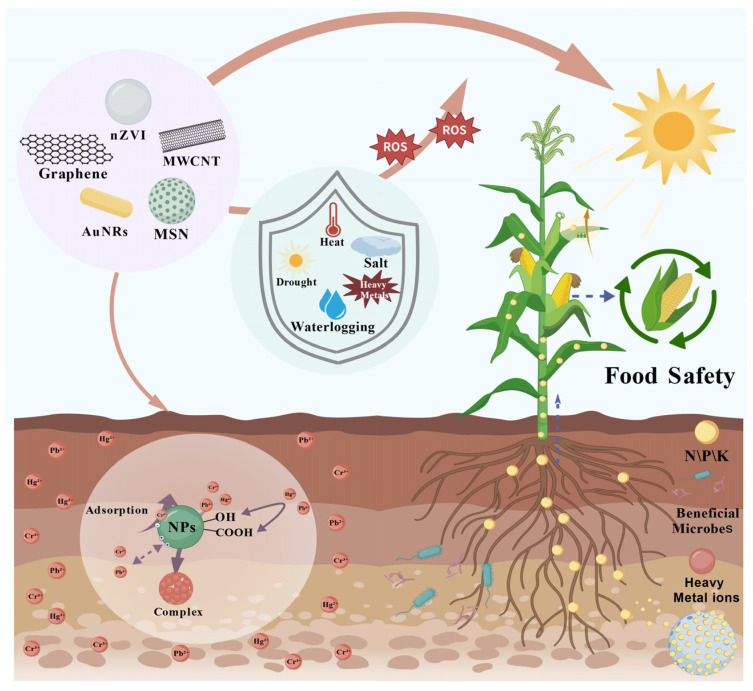
Schematic illustration of the multifunctional roles of nanomaterials (NMs) in agriculture. Multifunctional roles of nanomaterials (NMs) in alleviating plant abiotic stress and remediating soils. NMs regulate ROS dynamics, immobilize heavy metals (e.g., Pb^2+^, Cd^2+^), enable nutrient delivery, and enhance microbial recruitment and photosynthesis.

Climate-driven hydrological extremes, including droughts, intensified precipitation events, and temperature variability, induce cascading impacts on soil multifunctionality and agroecosystem resilience [21]. The altered abiotic stress regimes compromise plant physiological homeostasis, particularly through reactive oxygen species (ROS) overaccumulation, osmotic imbalance, and nutrient acquisition limitations [22]. Shah et al. (2011) [23] indicated peak sensitivity of rice to high temperatures during booting and flowering, with extreme heat causing floret sterility exacerbated by humid conditions. Li et al. (2021) [24] revealed that pre-growing-season drought and heat stress generally advance the onset date of vegetation dormancy across most regions of the Qinghai–Tibet Plateau. Extreme rainfall increases soil moisture, dilutes organic matter, elevates bioavailable metal fractions, and reduces Fe/Mn oxide-bound heavy metals, amplifying cumulative non-carcinogenic and carcinogenic risks by 10.1–188.3% [25]. Knight et al. (2024) [26] demonstrated that extreme events significantly alter functional gene abundances in 46% of soil microorganisms, affecting 8–61% of key functional categories. While plants possess innate defense mechanisms, developing non-toxic, sustainable technologies to enhance stress resilience remains crucial [27]. Remediation practices can improve soil health, alleviate crop stress, and strengthen resilience, thereby mitigating climate-induced yield fluctuations and food crises. Kumar et al. (2022) [27] revealed that biochar application regulates soil temperature and structure under heat stress, enhances porosity and water retention during drought, improves aeration and drainage in waterlogged conditions, and adsorbs salts to alleviate salinity stress. Nanoparticles (NPs) mitigate abiotic stress impacts—including drought, salinity, temperature extremes, heavy metal contamination, and light stress—through mechanisms such as altering plant hormone concentrations and gene expression [28]. Research by Song et al. (2021) [29] revealed that foliar-applied ZnO nanoparticles effectively mitigated low-temperature stress in hydroponic rice, resulting in enhanced dry biomass, restored chlorophyll accumulation, and improved oxidative stress responses. Soil application of nanoparticles enhances microbial or agriculturally beneficial microorganism functions, improving pollutant biodegradation or mitigating soil stress [30]. Thus, nanoparticles hold significant potential for countering soil–plant system damage from climate-driven extreme events. Nanobiotechnology, an interdisciplinary field converging nanotechnology and biotechnology, investigates the structure, function, and interactions of biological systems at the nanoscale (1–100 nm) [31]. It leverages the unique physical, chemical, and biological properties of nanomaterials to develop novel technologies for healthcare, agriculture, environmental management, and beyond (Figure 1).

In medicine, nanobiotechnology demonstrates significant application potential, particularly in drug delivery, diagnostic imaging, and regenerative medicine [32]. M13 bacteriophages serve as multivalent nanocarriers, displaying targeting peptides or antibodies to penetrate biological barriers for targeted cancer therapy and drug delivery across the blood–brain barrier [33]. Quantum dots (QDs) are widely used in cell analysis, imaging, and diagnostics due to their unique optical properties [34]. Wu et al. (2022) developed a CXCR4-inhibiting nanocomplex that improved liver fibrosis treatment by targeting Kupffer cells, hepatic stellate cells, and the extracellular matrix [35]. In agriculture, nanotechnology focuses on activating plant secondary metabolism to enhance stress adaptation. The high specific surface area and small size of nanoparticles improve tissue permeability, enabling development of nanofertilizers, nanopesticides, nanoweedicides, and nanosensors [28]. Slow-release nanofertilizers improve nutrient uptake and reduce environmental loss, facilitating synergistic delivery of agrochemicals [36]. Nanotechnology also offers solutions against fungicide resistance via nanoparticle-based antifungal agents [37]. Nanosensors exhibit exceptional sensitivity and selectivity for environmental monitoring; Kim et al. (2024) [38] highlighted the potential of intelligent odor-sensing nanosystems in environmental exposure detection and exhaled breath diagnostics. Furthermore, nanobiotechnology aids plant functional genomics and crop improvement. Cai et al. (2023) [39] developed a biocompatible mesoporous silica nanoparticle (MSN) platform for delivering siRNA into mature plant cells, enabling long-term multigene silencing and observable phenotypic changes. In materials engineering, nanomaterials enhance the mechanical and thermal properties of packaging, protecting food from external factors and preventing bacterial/fungal ingress [40]. In environmental science, nanobiotechnology has gained prominence for soil remediation. Synergistic interactions between nanomaterials and plant–microbe systems improve pollutant removal or stabilization efficiency. Green-synthesized Fe_3_O_4_ nanoparticles have demonstrated efficacy in extracting heavy metals, including Cr(VI), Cd, and Pb, from agricultural soils. Among the most extensively studied functional nanomaterials are nanoscale zero-valent iron (nZVI), carbon-based structures, and metal oxide nanoparticles (NPs) [41]. Sułowicz et al. (2023) [42] demonstrated that novel nanopesticide formulations alter non-target microbial exposure and shorten soil ecosystem recovery time compared to traditional captan@SiO_2_ nanopesticides.

Nanoparticles can exert short- or long-term effects on soil microorganisms. Metal oxides like TiO_2_ and ZnO alter microbial community structure and diversity [43]. Hou et al. (2021) [44] revealed that nematode cuticle collagen enhances nZVI migration, while secreted reductive biomolecules (e.g., L-cysteine) promote reductive dechlorination of pentachlorophenol (PCP), reducing nZVI dosage by 48.5% and increasing degradation efficiency 2.1-fold. Liu et al. (2020) [45] further found that nZVI increased rice yield by 47.1–55.0% and elevated PCP removal to 83.9–89.0%, while synergistic iron film formation and rhizospheric microorganisms reduced grain PCP content by 83.6–86.2%. While nanobiotechnology exhibits distinct advantages in remediating heavy metal-contaminated farmland and degrading organic pollutants in situ, research on its application for mixed contamination remediation remains limited.

Nanotechnology offers innovative solutions for agricultural stress through strong targeting capability, controlled release, and multi-functional integration [46]. Nanomaterials can achieve selective adsorption or directional transport via surface modification. He et al. (2024) [47] constructed a GABA-modified nanocarrier (PSI-GABA28) loaded with fluazinam, forming a core–shell Flu@PSI-GABA nanocomposite that exhibited long-distance transport to banana roots and stems, showing ~20-fold higher efficacy against Fusarium wilt than conventional formulations. Guo et al. (2025) [48] developed a microenvironment-responsive (pH, temperature, enzyme) eugenol nanobactericide using pectin-modified dendritic mesoporous silica nanoparticles (DMSNs), enabling precise pesticide release with minimal impact on non-target organisms. Wu et al. (2024) [49] synthesized λ-cyhalothrin-loaded poly (octyl acrylate) nanogels that enhanced aphid acute toxicity, extended residual activity, and reduced cytotoxicity.

Owing to their nanoscale dimensions, nanomaterials effectively penetrate biological barriers (cell walls, membranes). Their unique size effects enhance bioactivity, improve interaction efficiency with biological systems, and enable controlled release of loaded molecules [50]. Xu et al. (2021) [51] found that smaller MSNs were more readily absorbed and translocated in cucumber plants; pyraclostrobin-loaded carbon quantum dots-MSN (Pyr@M) exhibited a 3.5-fold higher upward translocation rate than free pyraclostrobin. Nanoparticle-rhizobacteria interactions can enhance soil productivity and plant performance; appropriate nanocompound addition improves soil microbial conditions, while agricultural use influences root exudates, rhizospheric microbiology, and soil health [50]. Lu et al. (2025) [52] revealed that iron-doped carbon quantum dots (Fe-CQDs) stimulated flavonoid production in wheat and regulated metal transporter genes (YSL, ABC, ZIP) to maintain ROS homeostasis under cadmium stress. Bueno et al. (2022) [53] reported that porous hollow silica nanoparticles (PHSNs) achieved 67% azoxystrobin loading with controlled release over days; nanocapsulated pesticides were absorbed and distributed more slowly in tomato plants. Haydar et al. (2023) [54] found that Fe–Mn-doped graphene quantum dots (FeMnO_4_@GQD) exhibited peroxidase-like activity, enhancing wheat seed germination, seedling growth, biomass, chlorophyll, carotenoids, sugars, proteins, and phenolics. Haider et al. (2024) [55] reported that seed priming with NCQDs enhanced corn seed germination and promoted overall plant growth, as reflected in increased shoot and root dry biomass. Diverse nanomaterials attract attention for their unique antimicrobial mechanisms, promising more effective and environmentally safe nanopesticides and herbicides [37,56,57]. Luo et al. (2021) [57] investigated a pesticide delivery system based on self-assembled degradable nanogels, which reduced UV degradation and aqueous exposure of cypermethrin, increasing its safety 9.33-fold; nanogel degradability facilitates exposure control and pollution reduction. Hussain et al. (2024) [58] showed that foliar spraying of Se-NPs reduced heavy metal accumulation in rice grains, increased Se, Zn, Fe, and protein levels, and decreased phytate, mitigating health risks from Cd-contaminated rice. Furthermore, nanobiotechnology leverages optical, electrical, or magnetic properties of nanomaterials for ultra-high-sensitivity detection]. Hu et al. (2025) [59] developed a non-destructive NIR-II fluorescent nanosensor for real-time detection of stress-related H_2_O_2_ signals in living plants; combined with an eXtreme Gradient Boosting algorithm training model, it identified four stress types (e.g., drought, high temperature) with >96.67% accuracy, <1 min response, and 0.43 μM detection limit. In summary, nanobiotechnology holds substantial potential for soil remediation and crop enhancement. However, further research and development are required to ensure its safe, efficient, and sustainable implementation [60].Current studies predominantly focus on pollutant adsorption by single-functional nanoparticles, largely overlooking the regulatory effects of dynamic root microenvironment responses and microbial community interactions on material efficacy. This review aims to elucidate the mechanisms through which nanobiotechnology enhances soil remediation and plant stress tolerance by integrating functional nanomaterials with plant–microbe systems to achieve sustainable restoration of contaminated and degraded soils.

## 2. Nanomaterials in Soil Remediation: Mechanisms and Applications

**Figure 2:**
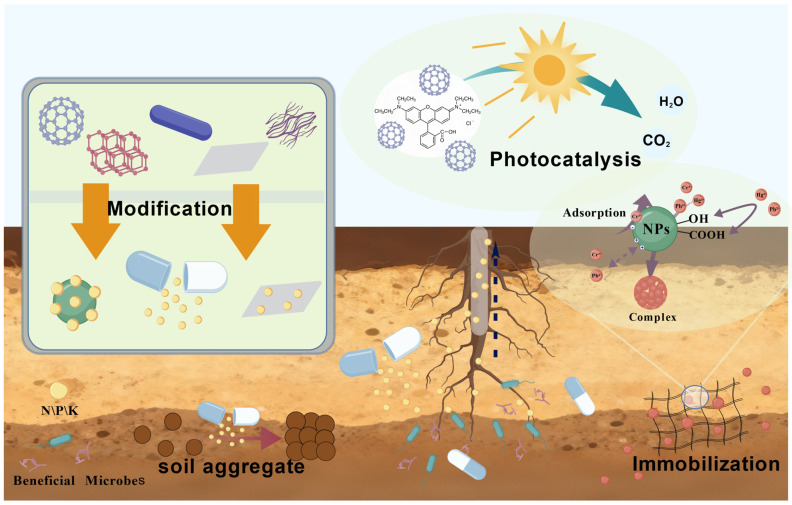
Schematic of soil remediation by nanomaterials (NMs). Remediation is achieved through: (i) photocatalytic decomposition of organics; (ii) heavy metal immobilization; (iii) controlled nutrient release; and (iv) enhanced soil aggregation.

Soil contamination and soil quality degradation represent critical challenges to the global ecological environment. Owing to their unique physicochemical properties, nanomaterials have demonstrated remarkable advantages in pollutant adsorption, catalytic degradation, and functional enhancement, providing a promising solution for soil remediation [61]. This section elaborates on the mechanisms and application of nanomaterials in pollutant removal and functional improvement, centered on their core mechanisms of action (Figure 2).

### 2.1. Contaminant Removal Strategies

#### 2.1.1. Adsorption and Immobilization

Nanomaterials exhibit an extremely high specific surface area, providing abundant adsorption sites that enable effective binding with heavy metal ions. Their primary mechanisms of action include physical adsorption, electrostatic adsorption, cation exchange, complexation, chemical precipitation, and cation-π interactions, emerging as a remediation strategy for the removal of heavy metals and organic pollutants [62]. The strong reducing capacity of nZVI/BC (nano-zero-valent iron/biochar) can effectively reduce most adsorbed heavy metals, thereby immobilizing them [63]. The surfaces of carbon-based nanomaterials are rich in functional groups (e.g., hydroxyl groups [-OH] and carboxyl groups [-COOH]), which form chemical bonds with heavy metal ions, capturing them through mechanisms such as physical adsorption, charge transfer, or ion exchange [64]. Biochar-based nanocomposites are formed through the incorporation of nanomaterials onto biochar matrices. These composites facilitate heavy metal immobilization through diverse mechanisms, such as physical adsorption, redox reactions, electrostatic interactions, coprecipitation, complexation, and ion exchange. Biochar electrostatic adsorption can fix heavy metal ions, and this effect is enhanced when combined with composite nanomaterials [65].

Polyakov et al. (2025) [66] optimized wheat straw biochar and further functionalized it by introducing metal–organic frameworks (MOFs). They found that coating biochar with MIL-100(Fe) increased its specific surface area sixfold to 419 m^2^·g^−1^, doubling its adsorption capacity for heavy metals in soil (142 mmol·kg^−1^ for Cu^2+^ and 156 mmol·kg^−1^ for Pb^2+^). Carbon nanotubes (CNTs), particularly multi-walled carbon nanotubes (MWCNTs), possess high adsorption capacity and can be used for the immobilization/stabilization of heavy metals. Correia et al. (2024) [67] demonstrated that multi-walled carbon nanotubes can reduce the mobility of heavy metals in soil even at very low concentrations (0.01% *w*/*w*). Pandey et al. (2021) [68] revealed that surface modification enhances the specific surface area of adsorbents, with functional groups dominating the chemisorption of heavy metals on modified adsorbents through electrostatic interactions and chelation/complexation. Liu et al. (2021) [69] observed that alkali-modified biochar (KRBC) exhibited higher specific surface area, more surface functional groups, and greater aromaticity compared to raw biochar (RBC) and acid-modified biochar (HRBC), making it the most effective in remediating Zn-contaminated soil. Nanoparticle-modified biochars derived from rice, wheat, corn straw, rice husks, sawdust, and wood chips can simultaneously immobilize arsenic (As) and cadmium (Cd), containing high levels of Fe_3_O_4_, iron oxides, and hydroxyl groups—ligand exchange dominates. As fixation, while ion exchange dominates Cd fixation [70].

Li et al. (2022) [71] introduced a novel biochar-supported phosphorus-doped ferrite (P-FH@BC) design, which enhances the passivation of lead (Pb) and cerium (Ce) in soil. Addressing soil heavy metal pollution requires attention to dosage, cost, and environmental sustainability, and recent applications of nanomaterials have provided new solutions. These advanced nanomaterials not only improve remediation efficiency but also emphasize environmental friendliness and recyclability. Compared to traditional remediation materials, magnetic nanomaterials can be recovered and reused using external magnetic fields. Owing to their pronounced surface effect, small size effect, and interfacial effect, they exhibit strong adsorption and immobilization capabilities for heavy metals [72]. Wang et al. (2024) [73] developed a UiO-66-Fe_3_O_4_ composite adsorbent with excellent magnetic properties and large specific surface area, showing high adsorption capacity for metal-EDTA complexes. Zirconium-oxygen coordination was identified as the key adsorption mechanism, enabling simple and low-cost recovery of heavy metals.

Cellulose nanomaterials, as templates, possess ideal characteristics: their tunable surfaces form covalent bonds, and they can be processed into diverse forms while retaining key properties. Their ease of synthesis and biodegradability have led to increasing applications in the environmental field [74]. Huang et al. (2024) [75] synthesized a carboxymethyl cellulose-nano zero-valent iron@biochar (CMC-nZVI@BC) composite, which demonstrated high remediation efficiency for hexavalent chromium (Cr (VI))-contaminated soil. It promoted the stabilization of Cr (VI), reducing its leachability and bioavailability. However, practical application studies of these new materials in soil remediation remain limited, necessitating further investigation into their dosage and toxicity.

#### 2.1.2. Catalytic Degradation

Soil organic contamination is one of the global environmental issues, primarily caused by pollutants such as pesticides, polycyclic aromatic hydrocarbons (PAHs), petroleum hydrocarbons, and industrial chemicals [76]. Under visible light irradiation, photocatalytic degradation of organic pollutants and adsorption/photocatalytic reduction of metal ions are effective methods for degrading organic molecules or immobilizing metal ions in situ. For low-concentration organic pollutants, photocatalytic degradation is the optimal approach [77]. The mechanism of organic pollutant photo-oxidation can be realized through two pathways: direct and indirect. The direct degradation mechanism involves the excitation of organic pollutants to a triplet excited state under visible light, injecting electrons into the conduction band of nanoparticles to generate radical cations. Dissolved oxygen then reacts with these electrons to produce superoxide anion radicals, which further generate hydroxyl radicals, primarily driving the oxidation of organic molecules. The indirect degradation mechanism occurs when semiconductor photocatalysts absorb photons with energy equal to or exceeding their band gap, exciting valence band photoelectrons to form electron-hole pairs. Hydroxyl radicals generated on the catalyst surface non-selectively attack nearby organic molecules, leading to their mineralization—an extent dependent on molecular structure and stability [78].

Carbon-based nanomaterials exhibit unique physical, chemical, and electronic properties. Multi-walled carbon nanotubes (MWCNTs) and single-walled carbon nanotubes (SWCNTs) can be used for removing organic and inorganic pollutants, while graphene serves as an adsorbent or component in photocatalytic composites [79]. Carbon-based nanomaterials themselves can act as photocatalysts, and their photocatalytic performance can be enhanced through modification with other materials [77]. When combined with clays, hybrid materials exhibit improved mechanical strength. Compared to pure clays, these nanocomposites demonstrate better molecular loading and controlled release capabilities, effectively removing pollutants in wastewater treatment and showing strong removal efficiency for per- and polyfluoroalkyl substances (PFAS) [80,81]. BC-nZVI (biochar-nano zero-valent iron) effectively removes organic pollutants such as phenols, dyes, and pesticides from water [82]. Toghan et al. (2023) [83] first investigated the composite film PVA/PANI/CGO (polyvinyl alcohol/polyaniline/carboxylated graphene oxide) and found that this nanocomposite film efficiently removes dye and antibiotic pollutants from wastewater.

Metal oxide nanomaterials (e.g., Fe_3_O_4_, TiO_2_) can generate free radicals (e.g., ·OH) via Fenton reactions, photocatalysis, or electrocatalysis, degrading organic pollutants into harmless small molecules [84]. Inorganic metal nanoparticles (mNPs) can also act as catalysts to chemically or photocatalytically reduce rhodamine B (RhB) [85]. When nZVI materials are combined with emulsion liquid membranes (ELMs), they more effectively catalyze the oxidative degradation of organic solvents in water [86]. Modification of Ag nanoparticles can suppress the recombination of photogenerated carriers in ZnO, and silver-modified photocatalytic materials also exhibit degradation capabilities for other pollutants such as tetracycline, polychlorinated biphenyls (PCBs), and phenol [87]. Doping with other metal ions enhances the surface activity of CeO_2_-based nanoparticles (NPs) during the degradation of organic dyes, phenols, amines, and aromatic nitro compounds. Doping introduces oxygen vacancies, further improving photocatalytic performance [88]. Alamelu et al. (2020) [89] synthesized highly dispersed sulfonated graphene nanosheets decorated with Ag and TiO_2_ nanoparticles (SGTAg) and demonstrated their efficacy as photocatalysts for degrading water-soluble cationic dyes (rhodamine B, Rh.B), anionic dyes (methyl orange, MO), and 4-nitrophenol (4-NP) under sunlight.

Immobilized on nanomaterials, enzymes serve as effective biocatalysts for the degradation of diverse organic pollutants, such as phenols, dyes, antibiotics, pesticides, and personal care products. This nano-confinement significantly improves the enzymatic stability, operational longevity, and resilience under fluctuating pH and temperature conditions [90]. Cellulose nanocrystals (CNCs) can also serve as stabilizers for catalysts to degrade organic pollutants or be incorporated into membranes for dye removal [91]. Practical application studies of these materials in soil remediation remain limited, necessitating further investigation.

### 2.2. Nutrient Delivery and Soil Health Enhancement

Global population growth has exacerbated agricultural productivity challenges and food security crises, with biotic stressors (e.g., fungi, bacteria) and abiotic stressors (e.g., salinity, drought) further contributing to yield losses. To address these issues, agricultural management employs diverse strategies to mitigate losses and reduce environmental impacts. Among these, exogenous protectants and nanotechnology have emerged as prominent green solutions. Nanoparticles—including titanium dioxide (TiO_2_), zinc oxide (ZnO), gold (Au), and silver (Ag)—applied in fertilizers, pesticides, and fungicides can enhance soil fertility, increase crop yields, and effectively mitigate both biotic and abiotic stresses [92].

#### 2.2.1. Nanofertilizers

Conventional fertilizers (e.g., urea) can experience losses of 50–70% via leaching, volatilization, or runoff after application to soil. Nanofertilizers [93], leveraging advanced nanotechnology, offer a high-efficiency and sustainable approach to crop fertilization by delivering plant nutrients in a controlled manner, ensuring gradual nutrient release over extended periods and thus providing stable supplies of essential elements to plants [94]. Compared to conventional fertilizers, nanofertilizers may enhance nutrient use efficiency (NUE) by up to 30% and increase crop yields by 20% [95]. Slow-release nanofertilizers (SRNFs) and controlled-release nanofertilizers (CRNFs) enable nutrient delivery through diverse mechanisms, including targeted nanoscale delivery, encapsulation for controlled release, and incorporation into organic bio-polymer matrices [96]. SRNFs release nutrients more slowly than conventional nanoparticles. Li et al. (2025) [97] developed a novel polyvinyl alcohol/starch (PVA/ST)-encapsulated zinc oxide (ZnO) nanoparticle-coupled biochar-based slow-release fertilizer (PVA/ST-Zn-BSRF). Field trials demonstrated that PVA/ST-Zn-BSRF increased wheat yield by up to 87.5%, significantly improved soil organic matter content, and generated higher wheat yields with an additional profit of USD 615.89 per hectare, confirming its potential to enhance agricultural productivity and sustainability. Maduwanthi et al. (2025) [98] incorporated nanoparticles (NPs) into a sodium alginate and carboxymethyl cellulose matrix, followed by ultrasonication and cross-linking with Ca^2+^ ions to form nanohybrids. Plant trials showed that this slow-release fertilizer significantly increased yields compared to plants treated with NPK alone. Sharma et al. (2020) [99] prepared a chitosan nanofertilizer co-encapsulated with copper and salicylic acid (SA), which slowly and sustainably supplied SA and Cu to plants. CRNFs are coated materials with controlled nutrient release rates and more predictable release patterns [96]. Timilsina et al. (2025) [100] found that chitosan-coated ZnO and silver (Ag) nanoparticles slowed changes in total soluble solids (TSS) and total titratable acidity (TTA), delaying papaya fruit ripening. Treated fruits exhibited reduced physiological weight loss and better firmness retention compared to the control group.

Tay et al. (2025) [101] loaded urea into cellulose nanoparticles (CNPs) derived from waste paper and experimentally verified its sustained urea release across diverse media, including frequently irrigated soil and water. Ghribi et al. (2025) [102] functionalized calcium phosphate nanoparticles (hydroxyapatite (HAP) and amorphous calcium phosphate (ACP)) as urea-loaded nanofertilizers to enable controlled co-release of phosphorus and nitrogen. Greenhouse experiments with Zea mays (maize) confirmed that ACP-urea treatment increased dry biomass and relative chlorophyll content. Latha et al. (2023) [103] loaded nitrogen sources into chitosan/lignin nanocomposite fertilizers (lignorea) composed of spherical lignin nanoparticles (LNPs), chitosan, and cross-linkers. These nitrogen-loaded nanofertilizers contained 30–35% total nitrogen, slowly released over 15 days in soil, and fixed/released nitrogen in a controlled manner.

While nanofertilizers demonstrate superior performance over conventional counterparts through controlled nutrient release, increased crop yield, and minimized environmental footprint, their excessive use poses a risk of phytotoxicity [104]. Their optimal application rates, potential toxicity, and long-term ecological effects require further investigation [105,106].

#### 2.2.2. Role of Nanomaterials in Improving Soil Structure, Water Retention, and Microbial Activity

Nanomaterials, characterized by high reactivity, mobility, and efficacy, play a critical role in soil remediation [107]. In soil environments, nanomaterials interact with organic or inorganic ligands, potentially altering soil porosity, influencing soil water dynamics, and affecting aggregate stability and microbial activity [108]. By virtue of their extremely small particle size, nanomaterials can penetrate soil micropores, enhancing soil porosity and effectively improving soil structure. Nanobiochar, for instance, enhances soil quality, water retention capacity, and nutrient retention capability [109]. Chen et al. (2021) [110] demonstrated via column experiments that nanoscale biochar particles (NBCs) alter the distribution of soil pore structures, increase soil hydrophobicity, reduce soil particle surface energy, elevate saturated hydraulic conductivity, and decrease water-holding capacity. Nepal et al. (2024) [111] demonstrated that carbon nanomaterials exhibit potential to mitigate nutrient leaching in coarse-textured soils. Ngo et al. (2024) [112] revealed that cellulose nanofibers (CNFs), due to their hydrophilic molecular groups and morphological structure, can absorb water; adding 1% CNF increased soil water content by 98%. In their hydrated state, CNFs promote colloidal flocculation and bind with soil particles, increasing macroaggregate formation by 48% and 59% in Massa soil and paddy soil samples, respectively.

Nanoparticle introduction into soil environments impacts microbial communities [43,113]. The effects of nanomaterials on soil microbial communities are multifaceted, encompassing changes in total microbial biomass, microbial richness, and community composition [114]. Cheng et al. (2020) [115] found that modified carbon black (MCB) influences soil enzyme activity, alters the abundance of specific bacterial taxa, and increases microbial community richness by reducing the bioavailability of heavy metals. Zuo et al. (2023) [116] concluded through a meta-analysis that exposure to carbon nanomaterials (CNMs) significantly decreases soil microbial biomass carbon (MBC) while enhancing microbial diversity. Udomkun et al. (2024) [117] demonstrated via black bean pot experiments that nanobiochar (NBC) significantly elevates soil pH, moisture content (MC), and soil organic carbon (SOC). Over six weeks, 3% and 1% NBC treatments consistently increased SOC content and boosted the abundance of total plate count (TPC), phosphate-solubilizing bacteria (PSB), and nitrogen-fixing bacteria (NFB). However, existing studies primarily employ single-strain methods under pure culture conditions [118,119,120]. Field conditions are typically more complex, necessitating improved evaluation mechanisms. Under appropriate nanomaterial types and concentrations, their stimulatory effects on soil microbial activity can enhance soil ecosystem health, strengthen nutrient cycling and decomposition capacity, and ultimately improve soil health status and fertility.

## 3. Nanomaterials in Promoting Plant Growth

**Figure 3:**
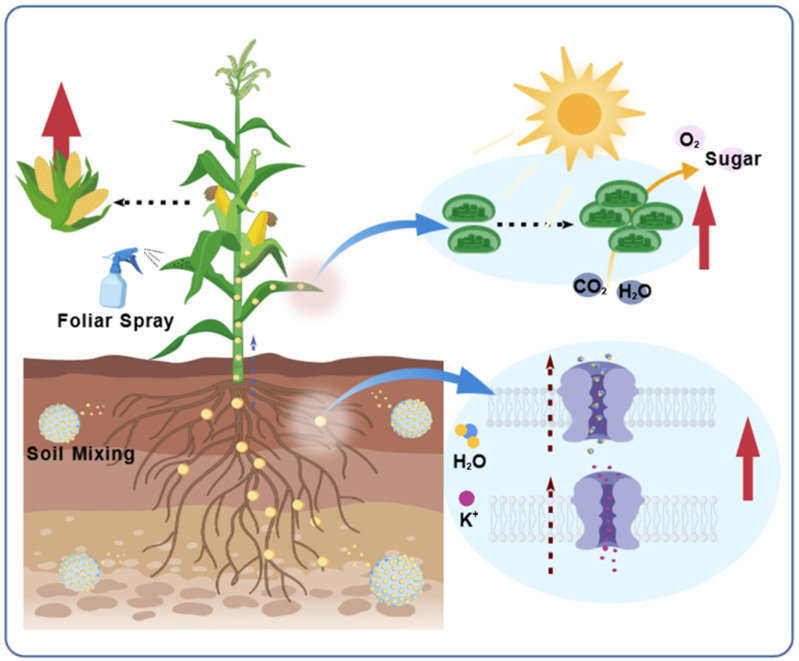
Nanomaterial (NM)-mediated promotion of plant growth. Applied via soil mixing or foliar spray, NMs enhance root uptake of water and ions by regulating channel proteins, and increase photosynthetic efficiency at the foliar level, collectively improving crop yield.

Nanomaterials, leveraging their unique physicochemical properties, demonstrate significant potential for promoting plant growth. Through diverse interaction pathways with plants, including enhanced nutrient uptake, improved photosynthetic efficiency, and regulated plant hormone homeostasis, they facilitate growth processes, thereby offering a novel avenue to augment crop yield and quality (Figure 3).

### 3.1. Enhanced Nutrient Uptake and Efficiency

Particle size is a critical factor influencing absorption and translocation, with nanoparticles (1–100 nm) demonstrating enhanced efficiency in penetrating plant tissues and being absorbed by plants [118]. Nanomaterials, leveraging their large specific surface area, further enhance contact with plant surfaces [121]. NPs can enter leaves via stomatal or cuticular pathways [50]. Research by Wang et al. (2025) [122] revealed that foliar-applied selenium nanoparticles (SeNPs) are effectively absorbed and systemically translocated within wheat plants. Compared to direct application, using P90H liposomes as nanocarriers significantly enhances selenium uptake in wheat [123]. Silica nanoparticles (SiNPs), as carriers, provide controlled release kinetics, improving nutrient absorption and utilization efficiency [124]. Mesoporous silica nanoparticles (MSNs), with unique structural features that accommodate various molecules, enable the delivery of chemical substances into plants [125]. Durgude et al. (2022) [126] found that foliar spraying of mesoporous silica micronutrient nanofertilizers increased micronutrient absorption in rice, alleviating “hidden hunger” in rice plants.

Nanoparticles can enter plant tissues via roots or aboveground organs [127]. Cheng et al. (2025) [128] showed that carbon nanosols activate root aquaporins to promote water and nutrient absorption, with single-cell transcriptomics revealing significant upregulation of related gene expression in epidermal and cortical cells. Accumulated potassium in cells correlates significantly with the fresh biomass of BY-2 cells; carbon nanoparticles (CNPs) upregulate potassium gene expression, enhancing K^+^ accumulation in BY-2 cells and improving plant growth [129].

Nanomaterials, as nutrient absorption forms, benefit plants [130]. Yin et al. (2024) [131] reported that soil application of 2.5 mg kg^−1^ Se-CQDs1 significantly promoted root growth, plant biomass, and fruit yield compared to the Na_2_SeO_3_ treatment (control). Slow and controlled release from nutrient carrier materials affects nutrient delivery rates to soil and edible crop parts. Slower nutrient delivery reduces loss, thereby improving root nutrient absorption efficiency [132]. Designing nanoparticles, nanocapsules, and nanoclays enables controlled nutrient release, better matching crop demands over extended periods [36]. Sigmon et al. (2021) [133] reported field-tested composite nanocapsules of polyhydroxyalkanoates (PHA) and calcium phosphate nanoparticles (Ca-P-NPs) in tomato cultivation, reducing phosphorus loss by 80%. Wang et al. (2024) [134] demonstrated that the application of potassium-based nanomaterials to soybeans achieved a utilization efficiency exceeding 80%, significantly outperforming that of conventional potassium fertilizers.

Nanomaterials offer an effective approach for agricultural nutrient delivery, addressing inefficiencies and environmental issues associated with traditional fertilization methods. However, attention must be paid to cost and biotoxicity.

### 3.2. Phytohormone Modulation and Root Development

Nanomaterials can serve as excellent carriers for plant hormone analogs such as auxins and cytokinins. Korpayev et al. (2021) [135] reported a significant promotion of root formation in apple rootstock microcuttings by employing chitosan and silver nanoparticles (AgNPs) as efficient nanocarriers for the auxins indole-3-acetic acid (IAA) and indole-3-butyric acid (IBA). Nano-silicon dioxide (SiO_2_) loaded with gibberellic acid (GA_3_) directly contributes to enhancing α-amylase activity through a nano-priming effect, thereby increasing the content of soluble sugars required to support seed germination and seedling growth [136].

Nanocarriers enable the precise delivery of hormones to specific plant tissues. Kokina et al. (2017) [137] developed a targeted delivery method using mesoporous gold/silicon dioxide (Au/SiO_2_) nanoparticles as carriers for plant hormones (e.g., auxins), which improved the incidence rate of somatic clonal variation in flax (*Linum usitatissimum* L.).

Nanomaterials interact with plant physiological processes by modulating hormone networks, regulating stomatal behavior, enhancing water use efficiency (WUE), and influencing chlorophyll synthesis and carbon fixation [138].

Xie et al. (2020) [139] revealed that graphene oxide (GO) modulates plant oxidative stress associated with IAA. Co-treatment with GO and IAA significantly regulated root length, adventitious root number, and the levels of IAA, cytokinin (CTK), and abscisic acid (ABA). Bhattacharya et al. (2023) [140] demonstrated that reduced graphene oxide (rGO) can act as a biostimulant to promote plant growth and serve as a nanodelivery carrier for exogenous application of plant growth regulators. Reduced graphene oxide (rGO) loaded with indole-3-acetic acid (IAA) significantly increased root length, stem length, and plant biomass in maize. Chitosan nanoparticles (CSNPs) more effectively promoted wheat growth at low concentrations by activating the IAA signaling pathway and enhancing chitosan adsorption onto seed surfaces [141].

Nanomaterials can also facilitate plant root growth and activity by recruiting beneficial microorganisms [46]. They modify the rhizospheric microenvironment to promote the colonization and activity of beneficial bacteria [142]. Jiao et al. (2023) [143] found that selenium nanoparticles (Se NMs) stimulated the growth of rhizospheric bacteria, particularly significantly increasing the relative abundance of *Streptomyces* and *Sphingomonas*, which enhanced root activity. The mechanisms underlying the interactions between nanomaterials, root microbiota, and root exudates exhibit complexity, and these processes demand further research attention.

### 3.3. Photosynthesis and Biomass Production

Plasmonic nanomaterials have garnered significant attention due to their unique optical and electronic properties. Plasmonic nanoparticles (e.g., gold and silver nanoparticles) can enhance the local electromagnetic field intensity around them through the surface plasmon resonance (SPR) effect, making chlorophyll molecules more susceptible to light absorption and thereby facilitating photochemical reactions, which provide additional energy for photosynthesis. In Al-Aaraji et al.’s (2025) [144] study, silver-modified ZnO (ZnO/Ag) nanoparticles were synthesized via a green method using mint extract. The localized surface plasmon resonance (LSPR) effect of silver significantly improved light absorption and charge carrier separation. Zhou et al. (2020) [145] employed non-noble plasmonic materials, including semiconductor nanocrystals with tunable plasmonic frequencies. Their research demonstrated that under infrared excitation, hot electrons were transferred on an ultrafast timescale (<50 fs) with an efficiency of 1.4%. Wolf et al. (2015) [146] found that plasmonic Cu_2−x_Se@ZnS core–shell nanoparticles hold application potential in scenarios requiring optical stability under complex chemical environments. Fan et al. (2025) [147] demonstrated that gold nanorod-modified MXene-enhanced phase-change composites increased light absorption efficiency by 29.7% compared to the original composites. Reddy et al. (2018) [148] observed that zinc oxide nanoparticles increased the content of photosynthetic pigments in coriander (*Coriandrum sativum*) grown in soil.

Nanomaterials can enhance the availability and absorption of nutrients by plants, contributing to improved plant growth, germination, and crop yield [149,150,151]. Sun et al. (2016) [152] found that mesoporous silica nanoparticles (MSNs) could be absorbed by plant roots and transported to aboveground tissues. Absorption of MSNs by wheat and lupin enhanced the accumulation of total leaf protein and chlorophyll, promoting photosynthesis and plant growth. Nepal et al. (2022) [153] reported that biochar nanomaterials (CNMs) at low to moderate concentrations improved lettuce growth, yield, and nutrient absorption. Zhao et al. (2024) [154] experimentally demonstrated that water-soluble cerium-doped carbon dots (50–150 mg·L^−1^) at low concentrations promoted lettuce yield and quality, as well as the accumulation of soluble sugars and soluble proteins. Nepal et al. (2023) [155] found that water-dispersible carbon nanomaterials (CNMs) at low to moderate application rates significantly enhanced lettuce growth, yield, leaf chlorophyll concentration, fluorescence, and photosynthetic activity. Pandey et al. (2024) [156] reported that supplementing soil with low doses (0.22 g/kg soil) of iron–carbon nanofibers/molybdenum-metal–organic frameworks increased chickpea fresh biomass and root-stem length while improving its nutritional quality. Zhao et al. (2021) [157] observed that the addition of carbon nanoparticles (CNPs) promoted the growth of maize plants in sandy soil, with an optimal application rate of 200 mg/kg. The application of nanofertilizers contributes to enhancing crop quality; however, attention must be paid to their potential biotoxicity and long-term environmental impacts. Research could focus on conducting life cycle assessments (LCA) to comprehensively evaluate these effects.

## 4. Nanomaterials in Enhancing Plant Stress Tolerance

**Figure 4:**
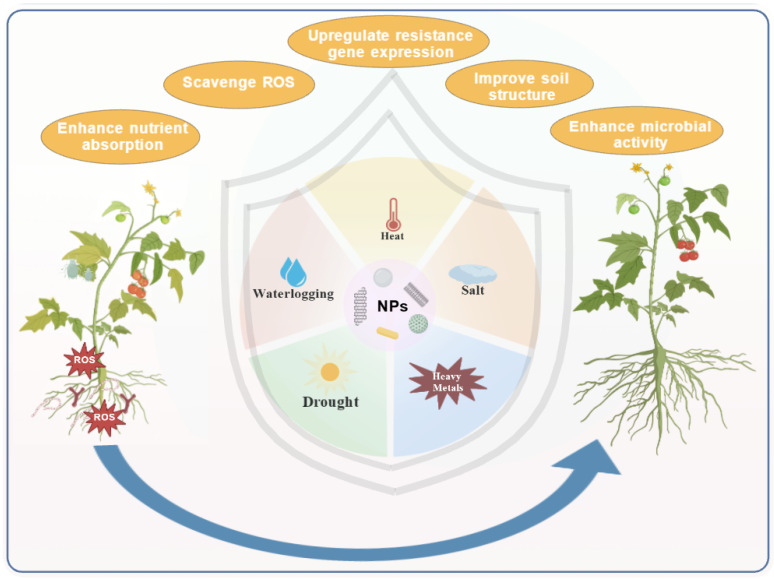
Plant abiotic stress alleviation by nanomaterials (NMs). NMs confer tolerance to heat, salt, and drought via integrated physiological and soil-level improvements, enabling recovery from stressed (left) to healthy growth (right).

Global climate change has intensified the threats posed by abiotic and biotic stresses—such as drought, salinity, heavy metal pollution, extreme temperatures, and pests/diseases—to crops. Traditional agricultural practices face challenges such as low efficiency, high costs, or environmental side effects when addressing complex stresses. Nanoparticles can enhance plant tolerance to stress [158]. By modifying environmental conditions, including soil physicochemical properties and microbial activity, nanomaterials actively promote plant growth and stress resistance (Figure 4). Alternatively, they regulate plant metabolism and gene expression to enhance antioxidant capacity and nutrient uptake, thereby strengthening plants’ adaptability to unfavorable conditions [159].

### 4.1. Abiotic Stress Mitigation

Common abiotic stresses include drought stress, heavy metal stress, salt stress, high-temperature stress, and biotic stress. In agriculture, nanomaterials are applied through various methods depending on their properties, including soil application, foliar spraying, and seed pretreatment. A meta-analysis by Chen et al. (2025) [160] revealed that nanomaterials significantly improved crop growth under drought conditions. Compared to root or foliar application, seed coating with nanomaterials demonstrated greater potential for crop protection.

To address drought stress, nanomaterials enhance plant water use efficiency through multiple pathways. Iron nanoparticles (Fe-NPs) improve plant stress tolerance under drought and salt conditions via a targeted delivery mechanism [161]. Silica nanoparticles (SiO_2_ NPs), nanohydrogels, and nanoclay composites enhance soil water retention capacity, thereby increasing the availability of water in the rhizosphere. Kim et al. (2022) [162] successfully synthesized nanocomposite hydrogels (NC) using laponite, potassium alginate (KA), and dimethylacrylamide (DMAAm). These hydrogels exhibited favorable swelling behavior in salt solutions under pH-dependent conditions, and plant growth experiments confirmed that soils amended with these hydrogels retained higher moisture content.

Excluding excess Na^+^ and Cl^−^ ions or sequestering them into vacuoles or older tissues represents a critical salt tolerance strategy [163]. Silica nanoparticles and cerium oxide nanoparticles have been used to enhance salt tolerance in various plant species [164]. Khan et al. (2024) [165] found that silicon nanoparticles (SiNPs) protected *Elymus sibiricus* (old wheatgrass) under drought and salt stress. Wang et al. (2025) [166] reported that SiNP treatment significantly increased K^+^ and Si content in tomato seedlings while reducing Na^+^ uptake. Desouky et al. (2024) [167] observed that foliar spraying of selenium nanoparticles (Se-NPs) on soybeans upregulated the GmHKT1gene, regulating K^+^/Na^+^ homeostasis in soybean plants under salt stress. Ghosh et al. (2024) [168] demonstrated that treatment with 5 g/L zinc-doped mesoporous silica nanoparticles (Zn-MSiNPs) increased the aboveground K^+^/Na^+^ ratio by 4.37-fold compared to a 200 mM NaCl treatment alone, effectively mitigating salt stress effects on wheat seedlings.

Nanomaterials reduce plant cell absorption of heavy metal ions through chelation or structural modification of heavy metal ions. For example, nano-titanium dioxide and nano-zinc oxide have shown promise in this regard [169]. Sharifan et al. (2019) [170] demonstrated via lettuce experiments that ZnONPs significantly reduced root accumulation of cadmium (Cd) and lead (Pb), with reductions of 49% and 81%, respectively. Recent studies on nanomaterials for mitigating abiotic stress are summarized in Table 1.

### 4.2. Biotic Stress Resistance

Plant biotic stress resistance is critical for maintaining crop yield and quality. Functioning as nanoscale elicitors, these materials bolster plant defense against biotic stressors such as pathogens and pests. This is achieved by priming innate immune responses and fostering beneficial microbe-plant symbiosis [46]. Nanopesticides, acting as inducers, activate plant defense genes to strengthen resistance to pathogens; for example, nanopesticides (silver and copper nanoparticles, NPs) are used for pathogen control and induction of systemic resistance (ISR) [185].

Jiang et al. (2022) [186] demonstrated that biosynthesized silver nanoparticles (AgNPs) inhibited *Pseudomonas syringaepv. Tabaci* (tobacco wildfire pathogen) through dual mechanisms: directly disrupting bacterial cells and inducing plant resistance in *Nicotiana benthamiana*. Feng et al. (2025) [187] proposed using nanoparticle pretreatment as a nano-mediated brassinosteroid (BR) hormone replacement therapy to trigger immune responses, thereby enhancing plant antiviral immunity. Results showed that leaves pretreated with zinc oxide nanoparticles (ZnONPs) exhibited accelerated antiviral capacity, with ZnONP-pretreated plants inducing systemic resistance (SR) to tobacco mosaic virus (TMV) via activation of the brassinosteroid pathway.

Nanomaterials can also be applied in plant disease management by directly inhibiting pathogens and modulating microbial communities [188]. Ouda et al. (2014) observed via microscopy that combined application of silver and copper nanoparticles caused damage to the hyphae and conidia of *Alternaria* and *Botrytis cinereafungi* [189]. Khan et al. (2023) [190] reported that biosynthesized copper oxide nanoparticles (CuONPs) exerted inhibitory effects against *Pectobacterium carotovorum* (carrot soft rot), *Phytophthora capsici* (pepper blight), and *Meloidogyne incognita* (southern root-knot nematode).

Targeted delivery nanocarriers respond to specific stimuli to release drugs at precise locations, improving therapeutic efficacy while minimizing off-target effects. Puangpathumanond et al. (2025) [191] developed surface ligand-engineered nanoparticles (SENDS) for targeted delivery to stomata, enhancing plant defense against invasive pathogens without disrupting natural stomatal function. Foliar application of SENDS encapsulating antimicrobial phytoalkaloids reduced colonization by the major crop pathogen *Xanthomonas campestrispv*. *campestrisby* 20-fold compared to non-targeted nanocarriers. Faraz et al. (2025) [192] synthesized chitosan-based nanoparticles loaded with imidacloprid; bioassays with cotton bollworms (*Helicoverpa armigera*) showed a 90% mortality rate within 48 h, superior to conventional pesticides (70% mortality) and exhibiting no phytotoxicity. Toxicity to non-target organisms was significantly reduced (e.g., mortality of *Daphnia magna* < 15%).

Paradoxically, certain nanoparticles can trigger an overaccumulation of reactive oxygen species (ROS) in plant tissues, which may lead to consequent oxidative damage [193].

### 4.3. Oxidative Stress Alleviation

When plants are exposed to various abiotic or biotic stresses, excessive reactive oxygen species (ROS)—including superoxide anion (O_2_^−^·), hydroxyl radical (·OH), and others—are generated in vivo. These ROS can attack plant cell membranes, proteins, nucleic acids, and other components, compromising cellular integrity [194]. Antioxidant nanoparticles, characterized by strong antioxidative capacity, can scavenge in vivo ROS and protect cells from oxidative damage. Examples include selenium nanoparticles (SeNPs) and fullerenes [195].

Gong et al. (2023) [196] designed carbon dot nanoenzymes (CDzymes) derived from glucose and histidine. Characterization results revealed their broad-spectrum antioxidative capacity, effectively scavenging reactive oxygen species (·OH, O_2_^−^·, H_2_O_2_), reactive nitrogen species (·NO, ONOO^−^), and stable free radicals (DPPH·, ABTS·^+^, PTIO·). Qin et al. (2024) [197]. experimentally demonstrated that molybdenum trioxide nanoparticles (MoO_3_ NPs) at low concentrations can mitigate oxidative damage in Solanum nigrum (black nightshade)

Keke et al. (2025) [198] found that silicon dioxide nanoparticles (SiNPs) improved wheat seed germination and seedling growth under drought by modulating antioxidant enzymes and mitigating oxidative damage. This protective effect was evidenced at 200 mg/L, where SiNPs treatment reduced lipid peroxidation and enhanced plasma membrane integrity. Drought alters the morphology of root epidermal cells; Sulaiman et al. (2024) [199] observed that SiNPs can reverse this effect: under drought conditions, antioxidant enzyme activity decreases, but SiNP application enhances it.

Different nanoparticles can upregulate or downregulate genes associated with antioxidant biosynthesis and plant hormone defense systems, thereby enhancing plant tolerance to salt stress by reducing ROS [200]. Chen et al. (2022) [201] conducted a joint transcriptomic and metabolomic analysis, showing that multi-walled carbon nanotubes (MWCNTs) regulate tyrosine (Tyr) and isoquinoline alkaloid biosynthesis in *Solanum nigrum* under heavy metal stress. This activation of the antioxidant defense system increased plant height and biomass, particularly in roots, without damaging the root tip epidermis.

Beyond boosting enzymatic systems, nanoparticles (NPs) further fortify the plant’s antioxidant capacity by elevating the levels of key non-enzymatic compounds, notably ascorbic acid (ASA) and glutathione (GSH), thereby significantly enhancing resilience to oxidative stress [202].

## 5. Environmental and Safety Considerations

### 5.1. Ecotoxicity and Long-Term Impacts

Some nanomaterials may exert adverse effects on soil microbial communities, reducing the diversity, biomass, activity, and functionality of soil microbial populations [151,203,204]. For example, graphene-based nanomaterials (GBNs) exhibit concentration- and time-dependent inhibitory effects on most tested bacteria and fungi [114]. Ouyang et al. (2021) [205] reported a slight inhibitory effect of multi-walled carbon nanotubes (MWCNTs) on the proliferation of the nitrogen-fixing bacterium *Azotobacter chroococcum*. Wu et al. (2020) [206] reported that MWCNTs (M50) significantly reduced the α-diversity of soil microbial functional genes. Ouyang et al. (2022) [207] demonstrated that metal–organic framework (MOF) materials, specifically MOF-199, exert toxic and activity-inhibitory effects on *Azotobacter vinelandii* (nitrogen-fixing bacteria). At 40 mg/L, MOF-199 significantly inhibited the growth of *A. vinelandiiand* caused cell death, posing environmental hazards and risks to nitrogen-fixing bacteria and nitrogen cycling in biogeochemical processes. Molybdenum nanoparticles (Mo-NPs) can inhibit soybean nitrogen fixation capacity [208].

Nanomaterials can accumulate in organisms and increase in concentration along the food chain. As they transfer from lower to higher trophic level organisms, their concentrations gradually rise, potentially causing greater harm to top predators [209,210,211]. Engineered nanomaterials (ENPs) deposit in aquatic plants and animals and transfer upward in the aquatic food chain, reaching three trophic levels [212]. Xiao et al. (2022) [213] found that silver nanoparticles (AgNPs), their sulfidation products (Ag_2_S-NPs), and dissolved products (Ag^+^) all accumulated in *Daphnia magna* (water fleas) and transferred to zebrafish through the food chain.

Nanoparticles applied in agriculture may reach other organisms, animals, and humans via the food chain [214]. Copper oxide nanoparticles (CuO NPs) can accumulate in humans and animals; when ingested, they are absorbed by the gastrointestinal tract and may cause various issues in vital organs [215]. ENMs can reach diverse organs, including the brain, and interact with glial cells and neurons, potentially inducing neurotoxicity [216]. Deng et al. (2019) [217] observed via mouse experiments that smaller-sized NPs exhibit more widespread organ distribution and longer circulation times. Wang et al. (2025) [122] conducted field trials of selenium nanoparticles (SeNPs) added to wheat throughout its life cycle, finding that SeNPs effectively delivered selenium to wheat grains, where it accumulated in ionic form rather than as nanoparticles, thereby avoiding the potential risk of direct human intake of nanoparticles. When applying nanomaterials for soil remediation and crop yield improvement, long-term impacts should be considered through life cycle assessment (LCA).

### 5.2. Regulatory Frameworks and Risk Assessment

The European Food Safety Authority (EFSA), as the core institution for food safety risk assessment in the European Union, is responsible for regulating the application of agricultural nanomaterials in food and feed. In 2018, it published the Scientific Guidelines for Risk Assessment of Nanomaterials in Food and Feed, which outlines four key requirements: (1) Material characterization must provide basic parameters (e.g., size distribution, shape, surface charge) and information on production impurities; (2) Exposure assessment must distinguish between “intentional addition” and “unintentional release” and quantify human/animal exposure doses; (3) Hazard identification should combine “top-down” and “bottom-up” approaches, with a focus on differences in toxicity between nanomaterials and their bulk counterparts; (4) Risk characterization must establish an “exposure-effect” model to define NOAEL (No Observed Adverse Effect Level) and LOAEL (Lowest Observed Adverse Effect Level). Additionally, EFSA emphasizes that the definition of nanomaterials requires dynamic adjustment—if materials exceed 100 nm but retain nanoscale properties, they remain subject to regulation [218].

In the United States, regulation of agricultural nanomaterials centers on the Federal Insecticide, Fungicide, and Rodenticide Act (FIFRA), with the Environmental Protection Agency (EPA) overseeing the registration and risk assessment of nanopesticides [219,220].

Nanomaterials may exhibit cytotoxicity at high concentrations; thus, toxicity analysis in normal cells is necessary before their biological application [221].

Ecotoxicity testing is foundational for assessing the environmental risks of nanomaterials. However, existing standards such as the OECD Guidelines for the Testing of Chemicalsare primarily designed for macroscopic substances and are not directly applicable to nanomaterials [222]. To address core challenges in nanomaterial ecotoxicity testing—including dispersion stability bias, insufficient sensitivity of traditional model organisms, and the lack of chronic toxicity assessment—the OECD Working Party on Manufactured Nanomaterials (WPMN) released the Guidance Document on Aquatic and Sediment Toxicological Testing of Nanomaterialsin 2021. This document complements the Nanotechnology—Environment, Health, and Safety Characterization Guidelines, collectively providing a unified framework for global nanomaterial ecotoxicity risk assessment [223,224].

Life cycle assessment (LCA), as a tool for evaluating the environmental impacts of nanomaterials in agriculture, requires consideration of all stages across their life cycle to comprehensively understand their environmental footprint [225]. LCA is a systematic approach to quantifying the environmental impacts of products, processes, or activities throughout their entire life cycle, from raw material extraction to final disposal or recycling [226]. Due to the unique physicochemical properties of nanomaterials, their application in agriculture—such as carriers for pesticides or fertilizers or for soil improvement—may result in complex environmental impacts [227]. Thus, conducting LCA on agricultural nanomaterials is critical for assessing their environmental sustainability.

### 5.3. Biodegradability and Sustainable Design

The development of plant/microbe-derived nanomaterials represents a critical pathway to addressing the environmental challenges posed by nanomaterials. Derived from natural biological resources, these nanomaterials exhibit excellent biodegradability and can be decomposed by microorganisms into harmless substances in the environment, thereby reducing environmental pollution [228].

Lignocellulosic biomass is recognized as a key renewable energy source and serves as a feedstock for preparing lignin nanoparticles (LNPs) [229]. LNPs are biodegradable and environmentally friendly, addressing limitations of bulk lignin such as heterogeneity and low water solubility [230,231]. Nanoscale lignin overcomes these challenges and demonstrates potential for biotechnological applications, offering a sustainable alternative to synthetic materials while possessing antibacterial and antioxidant properties [232].

Several studies have employed environmentally friendly methods to synthesize nanoparticles using lignin. For instance, Maršík et al. (2024) [233] utilized lignin as a renewable reducing agent and capping agent for silver nanoparticle synthesis. Rajput et al. (2025) [234] developed a green approach to produce stable, biocompatible lignin-coated silver nanoparticles (LS-AgNPs) using non-toxic lignosulfonate sodium as both a reducing and capping agent. Compared to pure silver nanoparticles, LS-AgNPs require minimal usage and exhibit significantly reduced toxicity in practical applications. Scopel et al. (2023) [235] leveraged elephant grass biorefining to generate cellulose and lignin nanoparticles.

Bacterial cellulose, a three-dimensional gel-like nanofiber structure, outperforms plant cellulose materials in terms of mechanical properties. It is non-toxic, biocompatible, and produced with low environmental resource consumption. Its sustainable supply and natural biodegradability in the environment minimize long-term waste impacts, aligning with environmental friendliness principles [236,237]. Additionally, cellulose has been functionalized with precious metal nanoparticles (e.g., silver and gold) for antimicrobial applications [238].

## 6. Challenges and Future Perspectives

**Figure 5:**
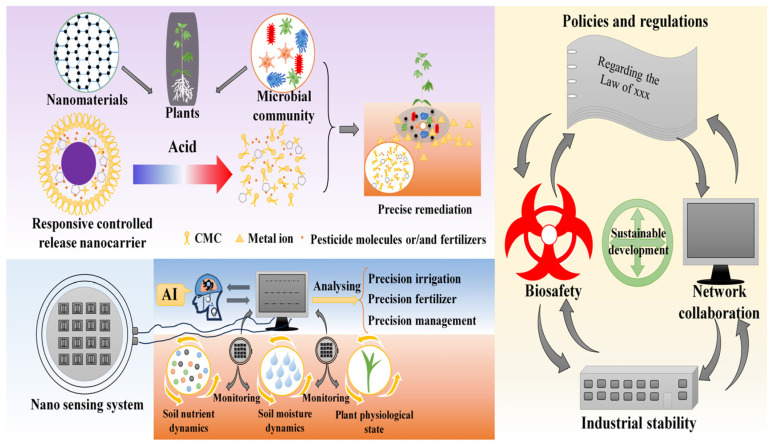
Challenges and prospects of nanomaterials in agriculture. Prospects include stimulus-responsive nanocarriers for precise remediation/nutrition and AI-integrated nanosensors for real-time field monitoring. Achieving these goals requires coordinated efforts in policy, biosafety, and sustainable development.

The synthesis of nanomaterials currently relies on methods such as lithography, chemical vapor deposition, sol–gel processing, and mechanochemistry, which are often energy-intensive, costly, and difficult to scale. Most novel nanomaterials remain confined to laboratory-scale production, limiting their widespread agricultural application. Future efforts should prioritize the use of low-cost raw materials, such as agricultural waste or industrial by-products, to enhance economic and environmental sustainability (Figure 5). The integration of life cycle assessment, green chemistry principles, and techno-economic analysis enables the identification of nanomaterial synthesis pathways that are both ecologically sound and economically viable [239,240].

Furthermore, most current agronomic applications of nanomaterials are limited to pot or small-scale field trials. Single-factor laboratory studies fail to capture the complex interactions in real farm environments, hindering accurate prediction of nanomaterial efficacy and complicating the translation of research into practical agricultural technologies [185].

A significant challenge is the limited understanding of the long-term behavior and environmental impact of nanomaterials. Key unresolved questions include their effects on soil physicochemical properties, microbial community structure, and potential for water pollution. These uncertainties highlight the need for intelligent nanosystems designed for controlled and targeted functions. For instance, pH-responsive nanocarriers enable precise delivery of agrochemicals based on spatial variations in soil conditions. Sun et al. (2023) [241] developed a smart pesticide delivery system (Tebu@ZnMOFs@CMC) that exhibits enhanced release under acidic or cellulase-rich conditions typical of disease sites.

Similarly, Piroonpan et al. (2024) [242] synthesized pH-responsive chitosan-polyacrylic acid nanoparticles for nitrogen fertilizer encapsulation, suitable for sandy soil improvement. Ma et al. (2023) [243] reported a dual-stimuli-responsive system for controlled pesticide release under conditions mimicking rice sheath blight infection. Such precision approaches improve resource efficiency while minimizing environmental impact. Synergistic integration of nanomaterials with phytoremediation and microbial communities offers another promising direction. Nanomaterials can enhance plant uptake and degradation of pollutants. For example, Chen et al. (2021) [244] found that multi-walled carbon nanotubes improved the phytoremediation efficiency of Solanum nigrum under cadmium and arsenic stress by promoting plant growth and stimulating antioxidant activity.

In another study, functional carbon nanodots enhanced water hyacinth’s ability to remove heavy metals from water and improved plant tolerance to lead-cadmium composite stress [245].

The integration of nanosensors with artificial intelligence (AI) will enable real-time soil–plant feedback systems, forming a critical tool for future agriculture. Nanosensors can monitor soil parameters (e.g., moisture, nutrients, pollutants) and plant physiological status in real time. Kumar et al. (2021) [246] developed a zinc oxide-carbon nanotube sensor for detecting soil potassium level and later Kumar et al. (2024) [247]. designed a nanoenzyme sensor array that effectively distinguishes and detects multiple pesticides at trace concentrations In addition, the accurate identification and characterization of these nanomaterials are crucial for assessing their environmental impacts and remediation effectiveness. This typically requires the integration of multiple advanced analytical techniques to obtain information on their size, shape, crystal structure, surface chemistry, elemental composition, and distribution within complex soil matrices [248].

AI algorithms can analyze these data to support precise decision-making, such as adjusting irrigation or fertilization, thereby advancing precision agriculture and improving crop yield and quality.

Overall, nanotechnology applications in agriculture align strongly with sustainable development goals. Intelligent nanosystems and synergistic approaches can reduce chemical inputs while maintaining productivity and minimizing environmental pollution. AI-driven monitoring supports rational resource use, protecting soil and water resources and promoting agricultural ecosystem stability. The large-scale application of agricultural nanotechnology requires not only technological innovation but also supportive policies, interdisciplinary collaboration, and global governance frameworks. Current challenges—including environmental and biosafety concerns, and regional disparities in application standards, necessitate policy regulation and international cooperation. Responsible development demands a “science-policy-industry” network to facilitate the safe and sustainable translation of nanotechnologies from lab to field [249,250].

International initiatives illustrate this trend. The European Union’s “Soil Mission” under the Green Deal identifies nanomaterials as key tools for soil restoration [251]. Projects such as “Prep Soil” integrate stakeholder feedback to establish pilot-to-scaling models [252], while the “NANOREM project” brings together materials scientists, soil scientists, and farmers to study silica nanoparticles’ effects on clay soils and monitor their environmental behavior [253]. In the United States, the USDA’s Nanoscale Agriculture Research Program supports industry-university collaborations to evaluate nanomaterial release, migration, and ecotoxicological impacts, systematically assessing long-term risks while promoting technological application [254].

Brazil’s Embrapa focuses on developing nanoscale slow-release fertilizers adapted to tropical conditions and accessible to smallholder farmers [255].

The complexity of agricultural nanotechnology necessitates deep interdisciplinary integration. From material synthesis to field application, and from environmental assessment to policy design, expertise from materials science, agronomy, ecology, toxicology, economics, and policy science must be combined. For example, developing pH-responsive nanofertilizers requires materials scientists to optimize carrier sensitivity, agronomists to validate performance in crop rhizospheres, ecologists to assess long-term soil microbial impacts, and economists to evaluate cost-effectiveness. This collaborative, full-chain approach—from laboratories to greenhouse to field—is essential for developing feasible, safe, and sustainable nano-agricultural solutions.

## 7. Conclusions

Nanotechnology plays a pivotal role in soil remediation by efficiently degrading organic pollutants, removing heavy metals, and improving soil quality. In sustainable agriculture, it promotes plant growth, enhances crop yield and quality, strengthens plant stress resistance, and demonstrates enormous transformative potential, offering new ideas and approaches to address global food security and environmental challenges. While advancing the application of nanotechnology in agriculture, it is critical to prioritize its environmental risks and biosafety and strengthen environmental management. Only by balancing innovation with environmental stewardship can we ensure that nanotechnology delivers agricultural benefits without compromising ecological integrity. Furthermore, the application of nanotechnology in agriculture intersects multiple disciplinary fields. A profound understanding of the mechanisms, environmental fate, and safety profiles of nanomaterials demands a concerted interdisciplinary effort. Such collaboration is imperative to overcome existing scientific hurdles and to guide the safe, effective, and responsible integration of nanotechnology in agricultural practices. This effort will contribute to achieving sustainable agricultural development.

## Figures and Tables

**Table 1:** Recent studies on nanomaterials for mitigating abiotic stress.

Abiotic Stress Type	Conditions	Nanomaterial Type	Application Method	Concentration	Plant Species	Cultivation Method	Effects	Duration from Treatment to Harvest	References
Drought stress		CeO_2_	Foliar spraying	100 mg/L	Mung bean	Pot cultivation	Significantly reduced damage	7–21 d	[171]
		ZnO	Foliar spraying	50–150 ppm	Wheat	Pot cultivation	Significantly increased weights	15 d	[172]
Cold stress		ZnO	Foliar spraying	25–100 mg/L	Rice	Hydroponic cultivation	Significantly alleviated negative impacts	22 d	[29]
		ZnO	Foliar spraying	25–100 mg/L	Aromatic rice	Soil cultivation	significantly increased root dry weight,	20 d	[173]
Low-light stress	Light intensity reduced by 67%	SiO_2_	Foliar spraying	0–300 mg/L	Maize	Hydroponic cultivation	significantly reduced H_2_O_2_ content	13 d	[174]
Salt stress		Zn	Seed priming	0–100 ppm	Wheat	Pot cultivation	reducing osmotic stress and regulating growth	90 d	[175]
	NaCl	FeO	Foliar spraying	0–100 mg/kg	Peanut	Hydroponic cultivation	50 mg/L Significantly improved growth parameters	10 d	[176]
Heavy metal stress	Cd	Si	Foliar spraying	10 mM	Maize	Pot cultivation	Significantly enhanced agronomic and physiological traits	15 d	[177]
	Cd	Se	Foliar spraying	0–40 mg/L	Wheat	Pot cultivation	Se-NPs application alleviated negative impacts.	Post-tillering to flowering stage	[178]
	Cr	SiO_2_	Seed priming	400 mg/L	Brassica napus	Hydroponic cultivation	significantly reduced Cr accumulation in leaves/roots	7 d	[179]
	Cr	CeO_2_	Foliar spraying	0–50 mg/L	Sunflower	Pot cultivation	Significantly promoted plant growth and biomass yield	4 m	[180]
	As, Cd	ZnO	Soil mixing	100 mg/kg	Rice	Flooded pot cultivation	Significantly reduced total As content in roots and stems	45 d	[181]
Combined stresses	Saline-alkali stress, Cd	P(AA-co-AM)/XLG hydrogel	Soil mixing	1%, 5%	Suaeda salsa	Pot cultivation	5% treatment significantly improved Cd removal efficiency	60 d	[182]
	Cd, drought	Si	Foliar spraying	25–100 mg/L	Wheat	Pot cultivation	Promoted wheat growth and yield	110 d	[183]
	Cd, alkaline	CeO_2_	Soil mixing	0–1000 mg/kg	Wheat	Pot cultivation	beneficial to wheat growth	154 d	[184]

## Data Availability

No new data were created or analyzed in this study.

## References

[1] Li G., Xing J. (2020). The Present Situation of Soil Pollution in Agricultural Production and the Countermeasures. IOP Conf. Ser. Earth Environ. Sci..

[2] FAO (2018). Soil Pollution—A Hidden Reality.

[3] FAO (2015). Status of the World’s Soil Resources.

[4] Prăvălie R., Necula N., Borrelli P., Tişcovschi A., Săvulescu I. (2025). A Complex Spatial Inventory of Land Degradation and Desertification in Romania. Ecol. Indic..

[5] FAO, UNEP (2021). Global Assessment of Soil Pollution: Summary for Policymakers.

[6] He S., Wei Y., Yang C., He Z. (2022). Interactions of Microplastics and Soil Pollutants in Soil-Plant Systems. Environ. Pollut..

[7] Sharifmand M., Sepehr E., Rasouli-Sadaghiani M., Asri-Rezaei S., Rengel Z. (2024). Antibiotics Pollutants in Agricultural Soil: Kinetic, Sorption, and Thermodynamic of Ciprofloxacin. Heliyon.

[8] Aghili S., Golzary A. (2023). Greening the Earth, Healing the Soil: A Comprehensive Life Cycle Assessment of Phytoremediation for Heavy Metal Contamination. Environ. Technol. Innov..

[9] Wei K.H., Ma J., Xi B.D., Yu M.D., Cui J., Chen B.L., Li Y., Gu Q.B., He X.S. (2022). Recent Progress on In-Situ Chemical Oxidation for the Remediation of Petroleum Contaminated Soil and Groundwater. J. Hazard. Mater..

[10] Sun Z., Zhao M., Chen L., Gong Z., Hu J., Ma D. (2023). Electrokinetic Remediation for the Removal of Heavy Metals in Soil: Limitations, Solutions and Prospection. Sci. Total Environ..

[11] Wu P., Wu X., Wang Y., Xu H., Owens G. (2022). Towards Sustainable Saline Agriculture: Interfacial Solar Evaporation for Simultaneous Seawater Desalination and Saline Soil Remediation. Water Res..

[12] Nair R.R., Russel J.G., Pradeep S., Ajay S.V., Krishnakumar B. (2020). A Novel Ex-Situ Bio-Remediation Process for Perchlorate Contaminated Soil. Chemosphere.

[13] Ren J., Song X., Ding D. (2020). Sustainable Remediation of Diesel-Contaminated Soil by Low Temperature Thermal Treatment: Improved Energy Efficiency and Soil Reusability. Chemosphere.

[14] Basta N.T., McGowen S.L. (2004). Evaluation of Chemical Immobilization Treatments for Reducing Heavy Metal Transport in a Smelter-Contaminated Soil. Environ. Pollut..

[15] Fu L., Zhang L., Dong P., Wang J., Shi L., Lian C., Shen Z., Chen Y. (2022). Remediation of Copper-Contaminated Soils Using *Tagetes patula* L., Earthworms and Arbuscular Mycorrhizal Fungi. Int. J. Phytoremediation.

[16] Wu C., Li F., Yi S., Ge F. (2021). Genetically Engineered Microbial Remediation of Soils Co-Contaminated by Heavy Metals and Polycyclic Aromatic Hydrocarbons: Advances and Ecological Risk Assessment. J. Environ. Manag..

[17] Hrapovic L., Sleep B.E., Major D.J., Hood E.D. (2005). Laboratory Study of Treatment of Trichloroethene by Chemical Oxidation Followed by Bioremediation. Hood Environ. Sci. Technol..

[18] Bai X., Wang Y., Zheng X., Zhu K., Long A., Wu X., Zhang H. (2019). Remediation of Phenanthrene Contaminated Soil by Coupling Soil Washing with Tween 80, Oxidation Using the UV/S2O82− Process and Recycling of the Surfactant. Chem. Eng. J..

[19] Guan C., Fu W., Zhang X., Li Z., Zhu Y., Chen F., Ji J., Wang G., Gao X. (2023). Enhanced Phytoremediation Efficiency of PHE-Contaminated Soil by Rape (*Brassica napus* L.) Assisted with PHE-Degradable PGPR through Modulating Rhizobacterial Communities. Ind. Crops Prod..

[20] Cao M., Lv W., Wang F., Ma S., Geng H., Li J., Gao Z., Xu Q., Guo J., Leng W. (2024). Foliar Application of Zinc Oxide Nanoparticles Alleviates Phenanthrene and Cadmium-Induced Phytotoxicity in Lettuce: Regulation of Plant–Rhizosphere–Microbial Long Distance. Environ. Sci. Technol..

[21] IPCC (2022). Climate Change 2022: Impacts, Adaptation and Vulnerability.

[22] Mittler R., Vanderauwera S., Suzuki N., Miller G.A.D., Tognetti V.B., Vandepoele K., Golley M., Shulaev V., Breusegem F.V. (2011). ROS Signaling: The New Wave?. Trends Plant Sci..

[23] Shah F., Huang J., Cui K., Nie L., Shah T., Chen C., Wang K. (2011). Impact of High-Temperature Stress on Rice Plant and Its Traits Related to Tolerance. J. Agric. Sci..

[24] Li P., Liu Z., Zhou X., Xie B., Li Z., Luo Y., Zhu Q., Peng C. (2021). Combined Control of Multiple Extreme Climate Stressors on Autumn Vegetation Phenology on the Tibetan Plateau under Past and Future Climate Change. Agric. For. Meteorol..

[25] Zhang L., Wang B., Wu W., Wang C., Cheng H., Duan X. (2024). Enhanced Health Risk of Soil Heavy Metal Exposure Following an Extreme Rainstorm under Climate Change. Sci. Total Environ..

[26] Knight C.G., Nicolitch O., Griffiths R.I., Goodall T., Jones B., Weser C., Langridge H., Davison J., Dellavalle A., Eisenhauer N. (2024). Soil Microbiomes Show Consistent and Predictable Responses to Extreme Events. Nature.

[27] Kumar A., Bhattacharya T., Mukherjee S., Sarkar B. (2022). A Perspective on Biochar for Repairing Damages in the Soil–Plant System Caused by Climate Change-Driven Extreme Weather Events. Biochar.

[28] Aguirre-Becerra H., Feregrino-Pérez A.A., Esquivel K., Perez-Garcia C.E., Vazquez-Hernandez M.C., Mariana-Alvarado A. (2022). Nanomaterials as an Alternative to Increase Plant Resistance to Abiotic Stresses. Front. Plant Sci..

[29] Song Y., Jiang M., Zhang H., Li R. (2021). Zinc Oxide Nanoparticles Alleviate Chilling Stress in Rice (*Oryza sativa* L.) by Regulating Antioxidative System and Chilling Response Transcription Factors. Molecules.

[30] Rajput V.D., Kumari A., Upadhyay S.K., Minkina T., Mandzhieva S., Ranjan A., Sushkova S., Burachevskaya M., Rajput P., Konstantinova E. (2023). Can Nanomaterials Improve the Soil Microbiome and Crop Productivity?. Agriculture.

[31] Zhang Y. (2005). Nanobiotechnology.

[32] Ali A., Kamran A. (2024). Nanobiotechnology in Health Sciences: Current Applications to Future Perspectives. Biol. Clin. Sci. Res. J..

[33] Chang C., Guo W., Yu X., Guo C., Zhou N., Guo X., Huang R.-L., Li Q., Zhu Y. (2023). Engineered M13 Phage as a Novel Therapeutic Bionanomaterial for Clinical Applications: From Tissue Regeneration to Cancer Therapy. Mater. Today Biol..

[34] Nabil M., Megahed F. (2023). Quantum Dot Nanomaterials: Preparation, Characterization, Advanced Bio-Imaging and Therapeutic Applications. J. Fluoresc..

[35] Wu P., Luo X., Sun M., Sun B., Sun M. (2022). Synergetic Regulation of Kupffer Cells, Extracellular Matrix and Hepatic Stellate Cells with Versatile CXCR4-Inhibiting Nanocomplex for Magnified Therapy in Liver Fibrosis. Biomaterials.

[36] Singh M., Goswami S.P., Ranjitha G., Sachan P., Sahu D.K., Beese S., Pandey S.K. (2024). Nanotech for Fertilizers and Nutrients-Improving Nutrient Use Efficiency with Nano-Enabled Fertilizers. J. Exp. Agric. Int..

[37] Javaid A., Hameed S., Li L., Zhang Z., Zhang B., Rahman M. (2024). Can Nanotechnology and Genomics Innovations Trigger Agricultural Revolution and Sustainable Development?. Funct. Integr. Genom..

[38] Kim C., Kang M.S., Raja I.S., Oh J.-W., Joung Y.K., Han D.-W. (2024). Current Issues and Perspectives in Nanosensors-Based Artificial Olfactory Systems for Breath Diagnostics and Environmental Exposure Monitoring. TrAC Trends Anal. Chem..

[39] Cai Y., Liu Z., Wang H., Meng H., Cao Y. (2023). Mesoporous Silica Nanoparticles Mediate SiRNA Delivery for Long-Term Multi-Gene Silencing in Intact Plants. Adv. Sci..

[40] Tamta S., Vimal V., Verma S., Gupta D., Verma D., Nangan S. (2024). Recent Development of Nanobiomaterials in Sustainable Agriculture and Agrowaste Management. Biocatal. Agric. Biotechnol..

[41] Andrade-Zavaleta K., Chacon-Laiza Y., Asmat-Campos D., Raquel-Checca N. (2022). Green Synthesis of Superparamagnetic Iron Oxide Nanoparticles with *Eucalyptus globulus* Extract and Their Application in the Removal of Heavy Metals from Agricultural Soil. Molecules.

[42] Sułowicz S., Markowicz A., Dulski M., Nowak A., Środek D., Borymski S. (2023). Assessment of the Ecotoxicological Impact of Captan@ZnO_35–45nm_ and Captan@SiO_2 20–30nm_ Nanopesticide on Non-Target Soil Microorganisms—A 100-Day Case Study. Appl. Soil Ecol..

[43] Sun C., Hu K., Mu D., Wang Z., Yu X. (2022). The Widespread Use of Nanomaterials: The Effects on the Function and Diversity of Environmental Microbial Communities. Microorganisms.

[44] Hou J., Hu C., White J.C., Yang K., Zhu L., Lin D. (2021). Nano–Zoo Interfacial Interaction as a Design Principle for Hybrid Soil Remediation Technology. ACS Nano.

[45] Liu Y., Wu T., White J.C., Lin D. (2020). A New Strategy Using Nanoscale Zero-Valent Iron to Simultaneously Promote Remediation and Safe Crop Production in Contaminated Soil. Nat. Nanotechnol..

[46] Sodhi G.K., Wijesekara T., Kumawat K.C., Adhikari P., Joshi K., Singh S., Farda B., Djebaili R., Sabbi E., Ramila F. (2025). Nanomaterials–Plants–Microbes Interaction: Plant Growth Promotion and Stress Mitigation. Front. Microbiol..

[47] He L., Xiao C., Zhu L., Deng W., Zhang Y., Li Y., Wu X., Wu H., Xu H., Jia J. (2024). GABA-Decorated Nanocarrier for Smart Delivery of Fludioxonil for Targeted Control of Banana Wilt Disease. J. Agric. Food Chem..

[48] Guo X., Li H., Li Z., Cui Z., Ma G., Nassor A.K., Guan Y., Pan X. (2025). Multi-Stimuli-Responsive Pectin-Coated Dendritic Mesoporous Silica Nanoparticles with Eugenol as a Sustained Release Nanocarrier for the Control of Tomato Bacterial Wilt. J. Nanobiotechnology.

[49] Wu T., Wang Y., Liu S., Zheng Z., He C., Yao W., Zhang C., Gu Y., Gao Y., Du F. (2024). Facile-Prepared Size-Controllable Nanogels for Enhancing Bidirectional Translocation, Control Efficiency, and Security of Nanopesticide. Adv. Funct. Mater..

[50] Wu H., Li Z. (2022). Nano-Enabled Agriculture: How Do Nanoparticles Cross Barriers in Plants?. Plant Commun..

[51] Xu Y., Xu C., Huang Q., Cao L., Teng F., Zhao P., Jia M. (2021). Size Effect of Mesoporous Silica Nanoparticles on Pesticide Loading, Release, and Delivery in Cucumber Plants. Appl. Sci..

[52] Lu Z., Li Y., Chen K., Chai S., Su G., Wu C., Sun M., Wang Y., Feng S., Hao M. (2025). Multi-Activity Ferruginated Carbon Quantum Dots Nanozyme Improves Wheat Seedling Growth and Cd Tolerance. Crop J..

[53] Bueno V., Gao X., Abdul Rahim A., Wang P., Bayen S., Ghoshal S. (2022). Uptake and Translocation of a Silica Nanocarrier and an Encapsulated Organic Pesticide Following Foliar Application in Tomato Plants. Environ. Environ. Sci. Technol..

[54] Haydar M.S., Ali S., Mandal P., Roy D., Roy M.N., Kundu S., Kundu S., Choudhuri C. (2023). Iron-Manganese Nanocomposites Doped Graphene Quantum Dots as Growth Promoter of Wheat and Its Biomimetic Activity. Biologia.

[55] Haider Z., Yang C., Ahmad I., Zia S., Javaid M.H., Rehman M., Yasin M.U., Ali B., Nana C., Gan Y. (2024). Bioderived Carbon Quantum Dots Boost Maize Growth and Photosynthesis by Augmenting UV Spectrum Absorption and Carbon Assimilation Regulatory Genes. J. Environ. Chem. Eng..

[56] Ali S., Ahmad N., Dar M.A., Manan S., Rani A., Alghanem S.M.S., Khan K.A., Sethupathy S., Elboughdiri N., Mostafa Y.S. (2023). Nano-Agrochemicals as Substitutes for Pesticides: Prospects and Risks. Plants.

[57] Luo J., Gao Y., Liu Y., Huang X., Zhang D., Cao H., Jing T., Liu F., Li B. (2021). Self-Assembled Degradable Nanogels Provide Foliar Affinity and Pinning for Pesticide Delivery by Flexibility and Adhesiveness Adjustment. ACS Nano.

[58] Hussain B., Yin X., Lin Q., Hamid Y., Usman M., Hashmi M.L.-R., Lu M., Imran Taqi M., He Z., Yang X. (2024). Mitigating Cadmium Exposure Risk in Rice with Foliar Nano-Selenium: Investigations through Caco-2 Human Cell Line In-Vivo Bioavailability Assay. Environ. Pollut..

[59] Hu H., Yuan H., Sun S., Feng J., Shi N., Wang Z., Liang Y., Ying Y., Wang Y. (2025). Machine Learning-Powered Activatable NIR-II Fluorescent Nanosensor for In Vivo Monitoring of Plant Stress Responses. Nat. Commun..

[60] Shah M.A., Shahnaz T., Masoodi J.H., Nazir S., Qurashi A., Ahmed G.H. (2024). Application of Nanotechnology in the Agricultural and Food Processing Industries: A Review. Sustain. Mater. Technol..

[61] Gong X., Huang D., Liu Y., Peng Z., Zeng G., Xu P., Cheng M., Wang R., Wan J. (2017). Remediation of Contaminated Soils by Biotechnology with Nanomaterials: Bio-Behavior, Applications, and Perspectives. Crit. Rev. Biotechnol..

[62] Fu T., Zhang B., Gao X., Cui S., Guan C.-Y., Zhang Y., Zhang B., Peng Y. (2023). Recent Progresses, Challenges, and Opportunities of Carbon-Based Materials Applied in Heavy Metal Polluted Soil Remediation. Sci. Total Environ..

[63] Zhong X., Lai Y., Wang X., Wang M., Han W., Zhang M., Ji H. (2024). Synthesis and Environmental Applications of Biochar-Supported Nano-Zero-Valent Iron Composites: A Review. Environ. Chem. Lett..

[64] Xie R., Jin Y., Chen Y., Jiang W. (2017). The Importance of Surface Functional Groups in the Adsorption of Copper onto Walnut Shell Derived Activated Carbon. Water Sci. Technol..

[65] Mazarji M., Bayero M.T., Minkina T., Sushkova S., Mandzhieva S., Bauer T.V., Soldatov A., Sillanpää M., Wong M.H. (2023). Nanomaterials in Biochar: Review of Their Effectiveness in Remediating Heavy Metal-Contaminated Soils. Sci. Total Environ..

[66] Polyakov V., Bauer T., Kirichkov M., Butova V., Gritsai M., Minkina T., Soldatov A., Kravchenko E. (2025). MOF-Biochar Nanocomposite for Sustainable Remediation of Contaminated Soil. Environ. Sci. Pollut. Res..

[67] Correia A.A.S., Rasteiro M.G. (2024). Effect of Multiwall Carbon Nanotubes and Surfactants Characteristics on Immobilization of Heavy Metals in Contaminated Soils. Discov. Appl. Sci..

[68] Pandey L.M. (2021). Surface Engineering of Nano-Sorbents for the Removal of Heavy Metals: Interfacial Aspects. J. Environ. Chem. Eng..

[69] Liu S., Xie Z., Zhu Y., Zhu Y., Jiang Y., Wang Y., Gao H. (2021). Adsorption Characteristics of Modified Rice Straw Biochar for Zn and In-Situ Remediation of Zn Contaminated Soil. Technol. Innov..

[70] Yasmin K., Hossain M.S., Li W.C. (2024). Simultaneous Immobilization Strategy of Anionic Metalloids and Cationic Metals in Agricultural Systems: A Review. Chemosphere.

[71] Li H., Jiang Q., Li R., Zhang B., Zhang J., Zhang Y. (2022). Passivation of Lead and Cerium in Soil Facilitated by Biochar-Supported Phosphate-Doped Ferrihydrite: Mechanisms and Microbial Community Evolution. J. Hazard. Mater..

[72] Zhang Y., Zhang Y., Wu A. (2024). Design and Construction of Magnetic Nanomaterials and Their Remediation Mechanisms for Heavy Metal Contaminated Soil. Sci. Total Environ..

[73] Wang X., Hussain A., Li Q., Ma M., Wu J., Deng M., Yang J., Li D. (2024). Core-Shell Design of UiO66-Fe_3_O_4_ Configured with EDTA-Assisted Washing for Rapid Adsorption and Simple Recovery of Heavy Metal Pollutants from Soil. J. Environ. Sci..

[74] Rahman A., Wang W., Govindaraj D., Kang S., Vikesland P.J. (2022). Recent Advances in Environmental Science and Engineering Applications of Cellulose Nanocomposites. Environ. Sci. Technol..

[75] Huang T., Li Y., Qian J., Liu S., Wang J. (2024). Remediation of Cr(VI)-Contaminated Soil by Double-Modified Nanoscale Zero-Valent Iron: Performance and Mechanism. J. Soils Sediments.

[76] Rani M., Shanker U. (2020). Environmental Nanotechnology Approaches for the Remediation of Contaminants. Bioremediation Technology.

[77] Liu Z., Ling Q., Cai Y., Xu L., Su J., Yu K., Wu J., Xu J., Hu B., Wang X. (2022). Synthesis of Carbon-Based Nanomaterials and Their Application in Pollution Management. Nanoscale Adv..

[78] Durodola S.S., Akeremale O.K., Ore O.T., Bayode A.A., Badamasi H., Olusola J.A. (2023). A Review on Nanomaterial as Photocatalysts for Degradation of Organic Pollutants. J. Fluoresc..

[79] Guerra F.D., Attia M.F., Whitehead D.C., Alexis F. (2018). Nanotechnology for Environmental Remediation: Materials and Applications. Molecules.

[80] Boruah J., Chowdhury D. (2022). Advances in Carbon Nanomaterial–Clay Nanocomposites for Diverse Applications. Minerals.

[81] Ali M.A., Thapa U., Antle J., Tanim E.U.H., Aguilar J.M., Bradley I.M., Aga D.S., Aich N. (2024). Influence of Water Chemistry and Operating Parameters on PFOS/PFOA Removal Using rGO-nZVI Nanohybrid. J. Hazard. Mater..

[82] Lu H., Wang X., Cong Q., Chen X., Li Q., Li X., Zhong S., Deng H., Yan B. (2024). Research Progress on the Degradation of Organic Pollutants in Water by Activated Persulfate Using Biochar-Loaded Nano Zero-Valent Iron. Molecules.

[83] Toghan A., Gouda M.H., Zahran H.F., Alakhras A.I., Sanad M.M., Elessawy N.A. (2023). Development of a New Promising Nanocomposite Photocatalyst of Polyaniline/Carboxylated Graphene Oxide Supported on PVA Film to Remove Different Ecological Pollutants. Diam. Relat. Mater..

[84] Khan Z.U.H., Gul N.S., Sabahat S., Sun J., Tahir K., Shah N.S., Muhammad N., Rahim A., Imran M., Iqbal J. (2023). Removal of Organic Pollutants through Hydroxyl Radical-Based Advanced Oxidation Processes. Ecotoxicol. Environ. Saf..

[85] Naseem K., Abrar E., Khalid A., Ismail M.A. (2024). Inorganic Nanoparticles as a Potential Catalyst for the Reduction of Rhodamine B Dye: A Critical Review. Inorg. Chem. Commun..

[86] Genuino H.C., Mazrui N., Seraji M.S., Luo Z., Hoag G.E. (2013). Green Synthesis of Iron Nanomaterials for Oxidative Catalysis of Organic Environmental Pollutants. New and Future Developments in Catalysis.

[87] Yu J., Yang Y., Sun F., Chen J. (2023). Research Status and Prospect of Nano Silver (Ag)-Modified Photocatalytic Materials for Degradation of Organic Pollutants. Environ. Sci. Pollut. Res..

[88] Madaan V., Mohan B., Bhankar V., Ranga R., Kumari P., Singh P., Sillanpää M., Kumar M., Solovev A.A., Kumar K. (2022). Metal-Decorated CeO_2_ Nanomaterials for Photocatalytic Degradation of Organic Pollutants. Inorg. Chem. Commun..

[89] Alamelu K., Jaffar Ali B.M. (2020). Ag Nanoparticle-Impregnated Sulfonated Graphene/TiO_2_ Composite for the Photocatalytic Removal of Organic Pollutants. Appl. Surf. Sci..

[90] Suresh R., Rajendran S., Khoo K.S., Soto-Moscoso M. (2022). Enzyme Immobilized Nanomaterials: An Electrochemical Bio-Sensing and Biocatalytic Degradation Properties Toward Organic Pollutants. Top. Catal..

[91] Zhang Y., Zhang Y., Xu W., Wu H., Shao Y., Han X., Zhou M., Gu P., Li Z. (2023). Preparation Methods of Cellulose Nanocrystal and Its Application in Treatment of Environmental Pollution: A Mini-Review. Colloid Interface Sci. Commun..

[92] Kale S.S., Chauhan R., Nigam B., Gosavi S., Chaudhary I.J. (2024). Effectiveness of Nanoparticles in Improving Soil Fertility and Eco-Friendly Crop Resistance: A Comprehensive Review. Biocatal. Agric. Biotechnol..

[93] Liang H., Shen P., Kong X., Liao Y., Liu Y., Wen X. (2020). Optimal Nitrogen Practice in Winter Wheat-Summer Maize Rotation Affecting the Fates of 15N-Labeled Fertilizer. Agronomy.

[94] Yadav A., Yadav K., Abd-Elsalam K. (2023). Nanofertilizers: Types, Delivery and Advantages in Agricultural Sustainability. Agrochemicals.

[95] Saurabh K., Prakash V., Dubey A.K., Ghosh S., Kumari A., Sundaram P.K., Jeet P., Sarkar B., Upadhyaya A., Das A. (2024). Enhancing Sustainability in Agriculture with Nanofertilizers. Discov. Appl. Sci..

[96] Haydar M.S., Ghosh D., Roy S. (2024). Slow and Controlled Release Nanofertilizers as an Efficient Tool for Sustainable Agriculture: Recent Understanding and Concerns. Plant Nano Biol..

[97] Li M., Zhao X., Cheng Y., Wu M., Dong C., Xiang H., Li Y., Cai Y., Zhang Z., Yu B. (2025). Zinc Oxide Nanoparticles Coupled Biochar-Based Slow-Release Fertilizer for Enhanced Nutrient Efficiency and Sustainable Agriculture. Ind. Crops Prod..

[98] Maduwanthi R.K., Munaweera I., Perera W.P.T.D. (2025). Nutrient Nanohybrids Based on Sodium Alginate and Carboxymethyl Cellulose Provide Enhanced Slow Release of Magnesium, Zinc and Copper. Chemical Papers.

[99] Sharma G., Kumar A., Devi K.A., Prajapati D., Bhagat D., Pal A., Raliya R., Biswas P., Saharan V. (2020). Chitosan Nanofertilizer to Foster Source Activity in Maize. Int. J. Biol. Macromol..

[100] Timilsina S., Adhikari R., Khatiwada P.P., Devkota G., Dahal K.C. (2025). *Ch*itosan-Based Zinc Oxide and Silver Nanoparticles Coating on Postharvest Quality of Papaya. SAARC J. Agric..

[101] Tay K.W.W., Chin S.F., Wasli M.E. (2025). Cellulose Nanoparticles as Controlled Release Nanocarriers for Urea. Waste Biomass Valorization.

[102] Ghribi S., Degli Esposti L., Steven B., Zuverza-Mena N., Yuan J., LaReau J.C., White J.C., Jaisi D.P., Adamiano A., Iafisco M. (2025). Functionalization of Amorphous and Crystalline Calcium Phosphate Nanoparticles with Urea for Phosphorus and Nitrogen Fertilizer Applications. J. Agric. Food Chem..

[103] Latha M., Subramanian K.S., Sundara Sharmila D.J., Raja K., Rajkishore S.K., Chitdeshwari T. (2023). Urea–Lignin/Chitosan Nanocomposite as Slow-Release Nanofertilizer. ACS Agric. Sci. Technol..

[104] Jithendar B., Kumar R., Rana N. (2024). Revolutionizing Crop Nutrition: Exploring Nano Fertilizers in Agriculture. Int. J. Plant Soil Sci..

[105] Kekeli M.A., Wang Q., Rui Y. (2025). The Role of Nano-Fertilizers in Sustainable Agriculture: Boosting Crop Yields and Enhancing Quality. Plants.

[106] Deng L., Wang A., Ma P., Zhu F., Du D., Okoye C.O., Chen C., Deng Q. (2025). Interaction between airway inflammation and gut microbiota dysbiosis caused by high temperatures (40 °C) and traffic-PM_2.5_ in mouse model. Environ. Res..

[107] Prabha V.V., Jayanthi M., Venkateshwar A. (2024). Nano Management Techniques for Soil Reclamation. J. Adv. Biol. Biotechnol..

[108] Xu Z., Long X., Jia Y., Zhao D., Pan X. (2022). Occurrence, Transport, and Toxicity of Nanomaterials in Soil Ecosystems: A Review. Environ. Chem. Lett..

[109] Brar B., Saharan B.S., Seth C.S., Kamboj A., Surkha, Bala K., Rajput V.D., Minkina T., Wong M.H., Kumar D. (2024). Nanobiochar: Soil and Plant Interactions and Their Implications for Sustainable Agriculture. Biocatal. Agric. Biotechnol..

[110] Chen X., Duan M., Zhou B., Cui L. (2021). Effects of Biochar Nanoparticles as a Soil Amendment on the Structure and Hydraulic Characteristics of a Sandy Loam Soil. Soil Use Manag..

[111] Nepal J., Xin X., Maltais-Landry G., Netto-Ferreira J.B., Wright A.L., He Z. (2024). Water Dispersible Carbon Nanomaterials Reduced N, P, and K Leaching Potential in Sandy Soils: A Column Leaching Study. Sci. Total Environ..

[112] Ngo A.T., Mori Y., Bui L.T. (2024). Effects of Cellulose Nanofibers on Soil Water Retention and Aggregate Stability. Environ. Technol. Innov..

[113] Iqbal B., Zhao T., Yin W., Zhao X., Xie Q., Khan K.Y., Zhao X., Nazar M., Li G., Du D. (2023). Impacts of soil microplastics on crops: A review. Appl. Soil Ecol..

[114] Cao H., Zhang X., Wang H., Ding B., Ge S., Zhao J. (2024). Effects of Graphene-Based Nanomaterials on Microorganisms and Soil Microbial Communities. Microorganisms.

[115] Cheng J., Sun Z., Li X., Yu Y. (2020). Effects of Modified Nanoscale Carbon Black on Plant Growth, Root Cellular Morphogenesis, and Microbial Community in Cadmium-Contaminated Soil. Environ. Sci. Pollut. Res..

[116] Zuo Y., Zeng W., Huang J. (2023). Effects of Exposure to Carbon Nanomaterials on Soil Microbial Communities: A Global Meta-Analysis. Land Degrad. Dev..

[117] Udomkun P., Chandi K., Boonupara T., Kaewlom P. (2024). Innovative Approaches: Exploring Nano-Biochar Technology’s Impact on Soil Properties, Alachlor Retention, and Microbial Populations. Environ. Technol. Innov..

[118] Chen X., Chu S., Chi Y., Wang J., Wang R., You Y., Hayat K., Khalid M., Zhang D., Zhou P. (2023). Unraveling the role of multi-walled carbon nanotubes in a corn-soil system: Plant growth, oxidative stress and heavy metal (loid) s behavior. Plant Physiol. Biochem..

[119] Dai Z.-C., Kong F.-L., Li Y.-F., Ullah R., Ali E.A., Gul F., Du D.-L., Zhang Y.-F., Jia H., Qi S.-S. (2024). Strong Invasive Mechanism of *Wedelia trilobata* via Growth and Physiological Traits under Nitrogen Stress Condition. Plants.

[120] Zhang H.Y., Goncalves P., Copeland E., Qi S.S., Dai Z.C., Li G.L., Wang C.Y., Du D.L., Thomas T. (2020). Invasion by the weed *Conyza canadensis* alters soil nutrient supply and shifts microbiota structure. Soil Biol. Biochem..

[121] Soni S.K., Dogra S., Sharma A., Thakur B., Yadav J., Kapil A., Soni R. (2024). Nanotechnology in Agriculture: Enhancing Crop Productivity with Sustainable Nano-Fertilizers and Nano-Biofertilizers. J. Soil Sci. Plant Nutr..

[122] Wang X., Hussain B., Xin X., Zou T., Huang X., Cheng L., Wu Z., Yang Y., Li Y., He Z. (2025). Fate and Physiological Effects of Foliar Selenium Nanoparticles in Wheat. ACS Nano.

[123] Viltres-Portales M., Sánchez-Martín M.-J., Boada R., Llugany M., Valiente M. (2024). Liposomes as Selenium Nanocarriers for Foliar Application to Wheat Plants: A Biofortification Strategy. Food Chem..

[124] Naaz H., Rawat K., Saffeullah P., Umar S. (2022). Silica Nanoparticles Synthesis and Applications in Agriculture for Plant Fertilization and Protection: A Review. Environ. Chem. Lett..

[125] Torney F., Trewyn B.G., Lin V.S.-Y., Wang K. (2007). Mesoporous Silica Nanoparticles Deliver DNA and Chemicals into Plants. Nat. Nanotechnol..

[126] Durgude S.A., Ram S., Kumar R., Singh S.V., Singh V., Durgude A.G., Pramanick B., Maitra S., Gaber A., Hossain A. (2022). Synthesis of Mesoporous Silica and Graphene-Based FeO and ZnO Nanocomposites for Nutritional Biofortification and Sustained the Productivity of Rice (*Oryza sativa* L.). J. Nanomater..

[127] Wang P., Lombi E., Zhao F.-J., Kopittke P.M. (2016). Nanotechnology: A New Opportunity in Plant Sciences. Trends Plant Sci..

[128] Cheng L., Qu Z., Chen Q., Wang L., Su H., Tao J., Lu P., Liang T., Zhang J., Cao P. (2025). Single-Cell Transcriptome Reveals Aquaporin-Mediated Carbon Nanosol-Induced Growth Promotion of Plants. Adv. Sci..

[129] Chen L., Yang J., Li X., Liang T., Nie C., Xie F., Liu K., Peng X., Xie J. (2020). Carbon Nanoparticles Enhance Potassium Uptake via Upregulating Potassium Channel Expression and Imitating Biological Ion Channels in BY-2 Cells. J. Nanobiotechnol..

[130] Faizan M., Karabulut F., Khan I., Akhtar M.S., Alam P. (2024). Emergence of Nanotechnology in Efficient Fertilizer Management in Soil. S. Afr. J. Bot..

[131] Yin K., Bao Q., Li J., Wang M., Wang F., Sun B., Sun B., Gong Y., Lian F. (2024). Molecular Mechanisms of Growth Promotion and Selenium Enrichment in Tomato Plants by Novel Selenium-Doped Carbon Quantum Dots. Chemosphere.

[132] Dutta S., Pal S., Panwar P., Sharma R.K., Bhutia P.L. (2022). Biopolymeric Nanocarriers for Nutrient Delivery and Crop Biofortification. ACS Omega.

[133] Sigmon L.R., Adisa I.O., Liu B., Elmer W.H., White J.C., Dimkpa C.O., Fairbrother D.H. (2021). Biodegradable Polymer Nanocomposites Provide Effective Delivery and Reduce Phosphorus Loss during Plant Growth. ACS Agric. Sci. Technol..

[134] Wang Q., Liao Y., Zhao W., Yi T., Jiang Y., Zhu G., Wang Q., Huang L., Chen F., Zhang P. (2024). Potassium-Based Nanomaterials Significantly Enhance Nutrient Utilization Efficiency and Promote High Crop Yields. Environ. Sci. Nano.

[135] Korpayev S., Karakeçili A., Dumanoğlu H., Ibrahim Ahmed Osman S. (2021). Chitosan and Silver Nanoparticles Are Attractive Auxin Carriers: A Comparative Study on the Adventitious Rooting of Microcuttings in Apple Rootstocks. Biotechnol. J..

[136] Chourasiya V.K., Nehra A., Shukla P.S., Singh K.P., Singh P. (2021). Impact of Mesoporous Nano-Silica (SiO_2_) on Seed Germination and Seedling Growth of Wheat, Pea and Mustard Seed. J. Nanosci. Nanotechnol..

[137] Kokina I., Jahundoviča I., Mickeviča I., Jermaļonoka M., Strautiņš J., Popovs S., Ogurcovs A., Sledevskis E., Polyakov B., Gerbreders V. (2017). Target Transportation of Auxin on Mesoporous Au/SiO_2_ Nanoparticles as a Method for Somaclonal Variation Increasing in Flax (*L. usitatissimum* L.). J. Nanomater..

[138] Dhiman V.K., Rana G., Dhiman V.K., Subbarayan R., Sharma M., Singh D., Jabir M., Ghotekar S., Chauhan A. (2025). Nanomaterials for Sustainable Agriculture: Plant Physiology and Environmental Resilience. Physiol. Mol. Plant Pathol..

[139] Xie L., Chen F., Du H., Zhang X., Wang X., Yao G., Xu B. (2020). Graphene Oxide and Indole-3-Acetic Acid Cotreatment Regulates the Root Growth of *Brassica napus* L. via Multiple Phytohormone Pathways. BMC Plant Biol..

[140] Bhattacharya N., Kochar M., Bohidar H.B., Yang W., Cahill D.M. (2023). Biologically Synthesized and Indole Acetic Acid-Loaded Graphene as Biostimulants for Maize Growth Enhancement. ACS Agric. Sci. Technol..

[141] Li R., He J., Xie H., Wang W., Bose S.K., Sun Y., Hu J., Yin H. (2019). Effects of Chitosan Nanoparticles on Seed Germination and Seedling Growth of Wheat (*Triticum aestivum L.*). Int. J. Biol. Macromol..

[142] Das D., Lah A. (2023). Utilizing Nanomaterials Linked with Plant Growth-Promoting Bacteria For Agricultural Advancements A Short Review. J. Adv. Zool..

[143] Jiao L., Cao X., Wang C., Chen F., Zou H., Yue L., Wang Z. (2023). Crosstalk between In Situ Root Exudates and Rhizobacteria to Promote Rice Growth by Selenium Nanomaterials. Sci. Total Environ..

[144] Al-Aaraji N.A.-H., Ghazi R.A., Heydaryan K., Kadhim S.A., Khojasteh H., Shaikhah D., Fini M.S. (2025). Plasmon-Enhanced Photocatalysis and Antimicrobial Activity of Green-Synthesized Ag-Decorated ZnO Nanoparticles Using *Mentha pulegium* Extract. Plasmonics.

[145] Zhou D., Li X., Zhou Q., Zhu H. (2020). Infrared Driven Hot Electron Generation and Transfer from Non-Noble Metal Plasmonic Nanocrystals. Nat. Commun..

[146] Wolf A., Härtling T., Hinrichs D., Dorfs D. (2015). Synthesis of Plasmonic Cu_2-*x*_Se@ZnS Core@Shell Nanoparticles. ChemPhysChem.

[147] Fan R., Chen G., Zheng N., Sun Z. (2025). Phase Change Composites Enhanced by Gold Nanorods Decorated MXene for Efficient Photothermal Conversion and Storage. Sol. Energy Mater. Sol. Cells.

[148] Reddy Pullagurala V.L., Adisa I.O., Rawat S., Kalagara S., Hernandez-Viezcas J.A., Peralta-Videa J.R., Gardea-Torresdey J.L. (2018). ZnO Nanoparticles Increase Photosynthetic Pigments and Decrease Lipid Peroxidation in Soil Grown Cilantro (*Coriandrum sativum*). Plant Physiol. Biochem..

[149] Otari S., Bapat V.A., Lakkakula J., Kadam U.S., Suprasanna P. (2024). Advancements in Bionanotechnological Applications for Climate-Smart Agriculture and Food Production. Biocatal. Agric. Biotechnol..

[150] Samal D.P.K., Sukla L.B., Bishoyi A.K. (2025). Biosynthesis of Phosphorus Nanoparticles for Sustainable Agroecosystems: Next Generation Nanotechnology Application for Improved Plant Growth. ACS Omega.

[151] Zhu S., Du H., Su F., Wang J., Meng Q., Liu T., Guo R., Chen Z., Li H., Liu W. (2023). Molecular cytogenetic analyses of two new wheat-rye 6RL translocation lines with resistance to wheat powdery mildew. Crop J..

[152] Sun D., Hussain H.I., Yi Z., Rookes J.E., Kong L., Cahill D.M. (2016). Mesoporous Silica Nanoparticles Enhance Seedling Growth and Photosynthesis in Wheat and Lupin. Chemosphere.

[153] Nepal J., Xin X., Maltais-Landry G., Wright A.L., Stoffella P.J., Ahmad W., He Z.L. (2022). Water-Dispersible Carbon Nanomaterials Improve Lettuce (*Latuca sativa*) Growth and Enhance Soil Biochemical Quality at Low to Medium Application Rates. Plant Soil.

[154] Zhao S., Li C., Wu C., Hu J., Zhang Z., Lei B., Li W., Hu C., Liu Y., Zheng M. (2024). Effects of Multifunctional Cerium-Doped Carbon Dots on Photosynthetic Capacity and Nutritional Quality of Lettuce. Environ. Sci. Nano.

[155] Nepal J., Xin X., Maltais-Landry G., Ahmad W., Pereira J., Santra S., Wright A.L., Ogram A., Stofella P.J., He Z. (2023). Carbon Nanomaterials Are a Superior Soil Amendment for Sandy Soils Than Biochar Based on Impacts on Lettuce Growth, Physiology and Soil Biochemical Quality. NanoImpact.

[156] Pandey K., Omar R.A., Verma N., Gupta G. (2024). Fe–Carbon Nanofiber-Modified Mo-MOF for the Controlled Release and Translocation of Micronutrients in Plants. Environ. Sci. Nano.

[157] Zhao F., Xin X., Cao Y., Su D., Ji P., Zhu Z., He Z. (2021). Use of Carbon Nanoparticles to Improve Soil Fertility, Crop Growth and Nutrient Uptake by Corn (*Zea mays L.*). Nanomaterials.

[158] Noreen S., Saleem M.H., Ali B., Khan K.A., Hafeez A., Javed M.A. (2024). Advancing Plant Resilience Against Microplastics and Metals Through Nanotechnology. BioNanoScience.

[159] Ping Y., Cao D., Hu J., Lin Y., Dang C., Xue D. (2024). The Application, Safety, and Challenge of Nanomaterials on Plant Growth and Stress Tolerance. Ind. Crops Prod..

[160] Chen L., Huang F., Liu J., Yang R., Hu Q., Li T., Zeng Y., Dai W., Qiu T., White J.C. (2025). Engineered Nanomaterials Enhance Crop Drought Resistance for Sustainable Agriculture. J. Agric. Food Chem..

[161] Razzaq S., Zhou B. (2025). Revolutionizing Crop Production with Iron Nanoparticles for Controlled Release of Plant Growth Regulators and Abiotic Stress Resistance. Plant Nano Biol..

[162] Kim S.M., Rhie Y.H., Kong S.M., Kim Y.S., Na Y.H. (2022). Synthesis of Nanocomposite Hydrogels for Improved Water Retention in Horticultural Soil. ACS Agric. Sci. Technol..

[163] Farooq M., Gogoi N., Hussain M., Barthakur S., Paul S., Bharadwaj N., Migdadi H.M., Alghamdi S.S., Siddique K.H. (2017). Effects, Tolerance Mechanisms and Management of Salt Stress in Grain Legumes. Plant Physiol. Biochem..

[164] Li Z., Zhu L., Zhao F., Li J., Zhang X., Kong X., Wu H., Zhang Z. (2022). Plant Salinity Stress Response and Nano-Enabled Plant Salt Tolerance. Front. Plant Sci..

[165] Khan I., Awan S.A., Rizwan M., Huizhi W., Ulhassan Z., Xie W. (2024). Silicon Nanoparticles Improved the Osmolyte Production, Antioxidant Defense System, and Phytohormone Regulation in *Elymus sibiricus* (L.) under Drought and Salt Stress. Environ. Sci. Pollut. Res..

[166] Wang S., Shen X., Guan X., Sun L., Yang Z., Wang D., Chen Y., Li P., Xie Z. (2025). Nano-Silicon Enhances Tomato Growth and Antioxidant Defense under Salt Stress. Environ. Sci. Nano.

[167] Desouky A.F., Desoukey S.F., Abdel-Aziz H.S.M., EL-kholy R.I., Hanafy M.S. (2024). Exogenous Application of Selenium Nanoparticles (Se-NPs) to Mitigate Salt Stress in Soybean-Evaluation of Physiological, Molecular and Biochemical Processes. J. Soil Sci. Plant Nutr..

[168] Ghosh D., Das T., Paul P., Dua T.K., Roy S. (2024). Zinc-Loaded Mesoporous Silica Nanoparticles Mitigate Salinity Stress in Wheat Seedlings through Silica-Zinc Uptake, Osmotic Balance, and ROS Detoxification. Plant Physiol. Biochem..

[169] Zhang Y., Qi G., Yao L., Huang L., Wang J., Gao W. (2022). Effects of Metal Nanoparticles and Other Preparative Materials in the Environment on Plants: From the Perspective of Improving Secondary Metabolites. J. Agric. Food Chem..

[170] Sharifan H., Ma X., Moore J.M., Habib M.R., Evans C. (2019). Zinc Oxide Nanoparticles Alleviated the Bioavailability of Cadmium and Lead and Changed the Uptake of Iron in Hydroponically Grown Lettuce (*Lactuca sativa L.* var. Longifolia). ACS Sustain. Chem. Eng..

[171] Maduraimuthu D., Alagarswamy S., Prabhakaran J., Karuppasami K.M., Venugopal P.B., Koothan V., Natarajan S., Dhashnamurthi V., Veerasamy R., Rathinavelu S. (2023). Drought Tolerance of Mungbean Is Improved by Foliar Spray of Nanoceria. Agronomy.

[172] Rukhsar-Ul-Haq, Kausar A., Hussain S., Javed T., Zafar S., Anwar S., Hussain S., Zahra N., Saqib M. (2023). Zinc Oxide Nanoparticles as Potential Hallmarks for Enhancing Drought Stress Tolerance in Wheat Seedlings. Plant Physiol. Biochem..

[173] Mai Y., Ren Y., Deng S., Ashraf U., Tang X., Duan M., Mo Z. (2024). Influence of ZnO Nanoparticles on Early Growth Stage of Fragrant Rice at Low Temperature (LT) Stress. J. Soil Sci. Plant Nutr..

[174] Li G., Ma Z., Zhang N., Li M., Li W., Mo Z. (2024). Foliar Application of Silica Nanoparticles Positively Influences the Early Growth Stage and Antioxidant Defense of Maize Under Low Light Stress. J. Soil Sci. Plant Nutr..

[175] Zafar S., Khan S., Ibrar D., Khan M.K., Hasnain Z., Mehmood K., Rais A., Gul S., Irshad S., Nawaz M. (2024). Application of Zinc Nanoparticles as Seed Priming Agent Improves Growth and Yield of Wheat Seedlings Grown under Salinity Stress by Enhanced Antioxidants Activities and Gas Exchange Attributes. Cereal Res. Commun..

[176] Zhang Y., Li L., Dai H., Kong X., Rahman M., Zhang B., Zhang Z., Zhou Y., Liu Q. (2025). Iron Oxide Nanoparticles (FeO-NPs) Mitigate Salt Stress in Peanut Seedlings by Enhancing Photosynthesis, Osmoregulation, and Antioxidant Activity. Plant Physiol. Biochem..

[177] Rahman S., Ahmad I., Nafees M. (2023). Mitigation of Heavy Metal Stress in Maize (*Zea mays* L.) through Application of Silicon Nanoparticles. Biocatal. Agric. Biotechnol..

[178] Nasirzadeh L., Kvarnheden A., Sorkhilaleloo B., Hervan E.M., Fatehi F. (2022). Foliar-Applied Selenium Nanoparticles Can Alleviate Soil-Cadmium Stress Through Physio-Chemical and Stomatal Changes to Optimize Yield, Antioxidant Capacity, and Fatty Acid Profile of Wheat (*Triticum aestivum L.*). J. Soil Sci. Plant Nutr..

[179] Ulhassan Z., Yang S., He D., Khan A.R., Salam A., Azhar W., Muhammad S., Ali S., Hamid Y., Khan I. (2023). Seed Priming with Nano-Silica Effectively Ameliorates Chromium Toxicity in Brassica Napus. J. Hazard. Mater..

[180] Ma J., Alshaya H., Okla M.K., Alwasel Y.A., Chen F., Adrees M., Hussian A., Hameed S., Shahid M.J. (2022). Application of Cerium Dioxide Nanoparticles and Chromium-Resistant Bacteria Reduced Chromium Toxicity in Sunflower Plants. Front. Plant Sci..

[181] Ma X., Sharifan H., Dou F., Sun W. (2020). Simultaneous Reduction of Arsenic (As) and Cadmium (Cd) Accumulation in Rice by Zinc Oxide Nanoparticles. Chem. Eng. J..

[182] Xu D., Zhang X., Liu J., Zhang Z., Qin C., Zhao Y., Wu G., Wu N., Xu W. (2025). Synergistic Remediation of Cadmium Pollution in Saline-Alkali Soil by Hydrogel and *Suaeda salsa*. ACS Appl. Mater. Interfaces.

[183] Adrees M., Khan Z.S., Rehman M.Z.U., Rizwan M., Ali S. (2022). Foliar Spray of Silicon Nanoparticles Improved the Growth and Minimized Cadmium (Cd) in Wheat under Combined Cd and Water-Limited Stress. Environ. Sci. Pollut. Res..

[184] Ayub M.A., Ahmad H.R., Zia ur Rehman M.Z., Waraich E. (2023). Cerium Oxide Nanoparticles Alleviates Stress in Wheat Grown on Cd Contaminated Alkaline Soil. Chemosphere.

[185] Zhang M., Ma W., Tao R., Fan Q., Zhang M., Qin D., Cao X., Li J., Xiong R., Huang C. (2024). Nanomaterials: Recent Advances in Plant Disease Diagnosis and Treatment. Nano Today.

[186] Jiang L., Xiang S., Lv X., Wang X., Li F., Liu W., Liu C., Ran M., Huang J., Xu X. (2022). Biosynthesized Silver Nanoparticles Inhibit *Pseudomonas syringae* pv. *tabaci* by Directly Destroying Bacteria and Inducing Plant Resistance in *Nicotiana benthamiana*. Phytopathol. Res..

[187] Feng H., Fan G., Liu Z., Zhou L., Wang X., Kang Z., Cai L. (2025). Nanomediated Stimulation: An Alternative to Brassinolide Hormone Replacement Therapy for Plant Resistance Activation. J. Agric. Food Chem..

[188] Ahmed T., Noman M., Gardea-Torresdey J.L., White J.C., Li B. (2023). Dynamic Interplay between Nano-Enabled Agrochemicals and the Plant-Associated Microbiome. Trends Plant Sci..

[189] Ouda S.M. (2014). Antifungal Activity of Silver and Copper Nanoparticles on Two Plant Pathogens, Alternaria alternata and *Botrytis cinerea*. Res. J. Microbiol..

[190] Khan M., Khan A.A., Parveen A., Min K., Yadav V.K., Khan A.U., Alam M. (2023). Mitigating the Growth of Plant Pathogenic Bacterium, Fungi, and Nematode by Using Plant-Mediated Synthesis of Copper Oxide Nanoparticles (CuO NPs). Green Chem. Lett. Rev..

[191] Puangpathumanond S., Chee H.L., Sevencan C., Yang X., Lau O.S., Lew T.T.S. (2025). Stomata-Targeted Nanocarriers Enhance Plant Defense against Pathogen Colonization. Nat. Commun..

[192] Faraz H.S., Darwish M.J., Shifeta N.T., Akram W. (2025). Development of Pesticide-Loaded Nanoparticles for Controlled and Targeted Crop Protection. J. Med. Health Sci. Rev..

[193] El-Saadony M.T., Saad A.M., Soliman S.M., Salem H.M., Desoky E.S.M., Babalghith A.O., El-Tahan M.T., Ibrahim O.M., Ebrahim A.A.M., El-Mageed T.A.A. (2022). Role of Nanoparticles in Enhancing Crop Tolerance to Abiotic Stress: A Comprehensive Review. Front. Plant Sci..

[194] Panda S.K., Gupta D., Patel M., Vyver C.V.D., Koyama H. (2024). Functionality of Reactive Oxygen Species (ROS) in Plants: Toxicity and Control in Poaceae Crops Exposed to Abiotic Stress. Plants.

[195] Rao M.J., Duan M., Zhou C., Jiao J., Cheng P., Yang L., Zheng B. (2025). Antioxidant Defense System in Plants: Reactive Oxygen Species Production, Signaling, and Scavenging During Abiotic Stress-Induced Oxidative Damage. Horticulturae.

[196] Gong J., Liu Q., Cai L., Yang Q., Tong Y., Chen X., Kotha S., Mao X., He W. (2023). Multimechanism Collaborative Superior Antioxidant CDzymes To Alleviate Salt Stress-Induced Oxidative Damage in Plant Growth. ACS Sustain. Chem. Eng..

[197] Qin M., Gong J., Zeng G., Cao W., Song B., Zhang Y., Fang S., Xu F., Wu Y. (2024). Molybdenum Trioxide Nanoparticles as Promising Stimulators for Facilitating Phytoremediation by Cd Hyperaccumulator *Solanum nigrum* L.. ACS Sustain. Chem. Eng..

[198] Keke L., Yiting L., Xiaohui Y., Yi Y., Junliang Y., Yunfeng C., Yongxing Z. (2025). Silica Nanoparticles Enhanced Seed Germination and Seedling Growth of Drought-Stressed Wheat by Modulating Antioxidant Enzymes and Mitigating Lipid Peroxidation. Environ. Sci. Nano.

[199] Sulaiman, Ahmad A., Noor Hassim M.F. (2024). Effects of Silica Nanoparticles on Morpho-Histological and Antioxidant Activities of Rice Seedlings under Drought Stress. S. Afr. J. Bot..

[200] Zia-ur-Rehman M., Anayatullah S., Irfan E., Hussain S.M., Rizwan M., Sohail M.I., Jair M., Ahmad T., Usman M., Alharby H.F. (2023). Nanoparticles Assisted Regulation of Oxidative Stress and Antioxidant Enzyme System in Plants under Salt Stress: A Review. Chemosphere.

[201] Chen X., Wang J., Wang R., Zhang D., Chu S., Yang X., Hayat K., Fan Z., Cao X., Ok Y.O. (2022). Insights into Growth-Promoting Effect of Nanomaterials: Using Transcriptomics and Metabolomics to Reveal the Molecular Mechanisms of MWCNTs in Enhancing Hyperaccumulator under Heavy Metal(loid)s Stress. J. Hazard. Mater..

[202] Singh K.M., Jha A.B., Dubey R.S., Sharma P. (2025). Nanoparticle-Mediated Mitigation of Salt Stress-Induced Oxidative Damage in Plants: Insights into Signaling, Gene Expression, and Antioxidant Mechanisms. Environ. Sci. Nano.

[203] Chen S., Teng Y., Luo Y., Kuramae E., Ren W. (2024). Threats to the Soil Microbiome from Nanomaterials: A Global Meta and Machine-Learning Analysis. Soil Biol. Biochem..

[204] Pan L., He F., Liang Q., Bo Y., Lin X., Javed Q., Ullah M.S., Sun J. (2023). Allelopathic effects of caffeic acid and its derivatives on seed germination and growth competitiveness of native plants (*Lantana indica*) and invasive plants (*Solidago canadensis*). Agriculture.

[205] Ouyang B., Yilihamu A., Liu D., Ouyang P., Zhang D., Wu X., Yang S.T. (2021). Toxicity and Environmental Impact of Multi-Walled Carbon Nanotubes to Nitrogen-Fixing Bacterium *Azotobacter chroococcum*. J. Environ. Chem. Eng..

[206] Wu F., You Y., Werner D., Wu F., You Y., Werner D., Jiao S., Hu J., Zhang X., Wan Y. (2020). Carbon Nanomaterials Affect Carbon Cycle-Related Functions of the Soil Microbial Community and the Coupling of Nutrient Cycles. J. Hazard. Mater..

[207] Ouyang B., Liu F., Liang C., Zhang J., Hu R., Yuan H., Hai R., Yuan Y., Wu X., Yang S.T. (2022). Toxicity and Activity Inhibition of Metal-Organic Framework MOF-199 to Nitrogen-Fixing Bacterium *Azotobacter vinelandii*. Sci. Total Environ..

[208] Zhou Y., Ma J., Yang J., Lv Z., Song Z., Han H. (2023). Soybean Rhizosphere Microorganisms Alleviate Mo Nanomaterials Induced Stress by Improving Soil Microbial Community Structure. Chemosphere.

[209] Uddin M.N., Desai F., Asmatulu E. (2020). Engineered Nanomaterials in the Environment: Bioaccumulation, Biomagnification and Biotransformation. Environ. Chem. Lett..

[210] Okeke E.S., Ezeorba T.P.C., Okoye C.O., Chen Y., Mao G., Feng W., Wu X. (2022). Analytical detection methods for azo dyes: A focus on comparative limitations and prospects of bio-sensing and electrochemical nano-detection. J. Food Compos. Anal..

[211] Han C., Xiao Y., Liu Z., Du D., Li M. (2023). Cascade amplifying aptasensor for positively correlated detecting OTA: Based on DNase I-assisted cyclic enzyme digestion and AgNPs@ gel-enhanced fluorescence. Food Control.

[212] Krishna D., Sachan H.K. (2021). Nano-Toxicity and Aquatic Food Chain. Advances in Science, Technology & Innovation.

[213] Xiao B., Yang R., Chen P., Yang J., Sun B., Wang K., Zhong T., Zhu L. (2022). Insights into the Lower Trophic Transfer of Silver Ions than Silver Containing Nanoparticles along an Aquatic Food Chain. Sci. Total Environ..

[214] Siddiqi K.S., Husen A. (2017). Plant Response to Engineered Metal Oxide Nanoparticles. Nanoscale Res. Lett..

[215] Sharma R., Sharma N., Prashar A., Hansa A., Lajayer B.A., Price G.W. (2024). Unraveling the Plethora of Toxicological Implications of Nanoparticles on Living Organisms and Recent Insights into Different Remediation Strategies: A Comprehensive Review. Sci. Total Environ..

[216] Ge D., Du Q., Ran B., Liu X., Wang X., Ma X., Cheng F., Sun B. (2019). The Neurotoxicity Induced by Engineered Nanomaterials. Int. J. Nanomed..

[217] Deng L., Liu H., Ma Y., Miao Y., Fu X., Deng Q. (2019). Endocytosis Mechanism in Physiologically-Based Pharmacokinetic Modeling of Nanoparticles. Toxicol. Appl. Pharmacol..

[218] EFSA Panel on Food Contact Materials, Enzymes and Processing Aids (CEP) (2021). Guidance on Risk Assessment of Nanomaterials to Be Applied in the Food and Feed Chain: Human and Animal Health. EFSA J..

[219] U.S. Environmental Protection Agency (EPA) (1947). Federal Insecticide, Fungicide, and Rodenticide Act (FIFRA). 7 U.S.C. §136. https://www.epa.gov/laws-regulations/summary-federal-insecticide-fungicide-and-rodenticide-act.

[220] U (2015). S. Environmental Protection Agency. Chemical Substances When Manufactured or Processed as Nanoscale Materials; TSCA Reporting and Recordkeeping Requirements. Fed. Regist..

[221] Ghosh S., Yadav P., Sankaranarayanan S., Bhatia D. (2023). Plant-Derived Nanomaterials for Targeted Biological Applications and Smart Agriculture. ChemistrySelect.

[222] Organisation for Economic Co-Operation and Development (OECD) OECD Guidelines for the Testing of Chemicals. https://www.oecd-ilibrary.org/environment/oecd-guidelines-for-the-testing-of-chemicals_72d77764-en.

[223] Organisation for Economic Co-Operation and Development (OECD) (2021). Guidance Document on Aquatic and Sediment Toxicological Testing of Nanomaterials. OECD Environment, Health and Safety Publications, Series on Testing and Assessment No. 317.

[224] (2022). Nanotechnologies—Guidance on Characterization of Engineered Nanomaterials for Environmental Health and Safety.

[225] Andersson K., Ohlsson T., Olsson P. (1994). Life Cycle Assessment (LCA) of Food Products and Production Systems. Trends Food Sci. Technol..

[226] Herrchen M., Klein W. (2000). Use of the Life-Cycle Assessment (LCA) Toolbox for an Environmental Evaluation of Production Processes. Pure Appl. Chem..

[227] Nizam N.U.M., Hanafiah M.M., Woon K.S. (2021). A Content Review of Life Cycle Assessment of Nanomaterials: Current Practices, Challenges, and Future Prospects. Nanomaterials.

[228] Alsaiari N.S., Alzahrani F.M., Amari A., Osman H., Harharah H.N., Elboughdiri N., Tahoon M.A. (2023). Plant and Microbial Approaches as Green Methods for the Synthesis of Nanomaterials: Synthesis, Applications, and Future Perspectives. Molecules.

[229] Shalma S., Shabbirahmed A.M., Haldar D., Patel A.K., Singhania R.R. (2023). Influence of Reaction Conditions on Synthesis and Applications of Lignin Nanoparticles Derived from Agricultural Wastes. Environ. Technol. Innov..

[230] Saxena A., Parveen F., Hussain A., Khubaib M., Ashfaque M. (2025). Exploring the Multifaceted Landscape of Lignocellulosic Biomass-Derived Nanocellulose and Nanolignin: Synthesis and Applications. Polym. Bull..

[231] Yadav V.K., Gupta N., Kumar P., Dashti M.G., Tirth V., Khan S.H., Yadav K.K., Islam S., Choudhary N., Algahtani A. (2022). Recent Advances in Synthesis and Degradation of Lignin and Lignin Nanoparticles and Their Emerging Applications in Nanotechnology. Materials.

[232] Yu X., Yang B., Zhu W., Deng T., Pu Y., Ragauskas A., Wang H. (2023). Towards Functionalized Lignin and Its Derivatives for High-Value Material Applications. Ind. Crops Prod..

[233] Maršík D., Thoresen P.P., Maťátková O., Masák J., Sialini P., Rova U., Tsikourkitoudi V., Christakopoulos P., Matsakas L., Jarošová Kolouchová I. (2024). Synthesis and Characterization of Lignin-Silver Nanoparticles. Molecules.

[234] Rajput S.K., Banerjee S., Sharma V., Ali S.W., Singh M.K., Shakyawar D.B. (2025). A Facile and Greener Approach for Synthesis of Lignin Capped-Silver Nanoparticles (LS-AgNPs) and Assessment of Their Antibacterial and Antioxidant Properties. J. Mol. Struct..

[235] Scopel E., Camargos C.H.M., Pinto L.O., Trevisan H., Ferreira E.S., Rezende C.A. (2023). Broadening the Product Portfolio with Cellulose and Lignin Nanoparticles in an Elephant Grass Biorefinery. Biofuels Bioprod. Biorefining.

[236] Wasim M., Mushtaq M., Khan S.U., Farooq A., Naeem M.A., Khan M.R., Salam A., Wei Q. (2020). Development of Bacterial Cellulose Nanocomposites: An Overview of the Synthesis of Bacterial Cellulose Nanocomposites with Metallic and Metallic-Oxide Nanoparticles by Different Methods and Techniques for Biomedical Applications. J. Ind. Text..

[237] Hu W., Chen S., Yang J., Li Z., Wang H. (2014). Functionalized Bacterial Cellulose Derivatives and Nanocomposites. Carbohydr. Polym..

[238] Agarwal C., Csoka L. (2018). Functionalization of Wood/Plant-Based Natural Cellulose Fibers with Nanomaterials: A Review. TAPPI J..

[239] Thangadurai D., Prakash L., Sangeetha J., Al-Tawaha A.R.M.S., David M., Islam S., Adetunji J.B. (2021). Sustainable Synthesis of Greener Nanomaterials: Principles, Processes, and Products. Handbook of Nanomaterials and Nanocomposites for Energy and Environmental Applications.

[240] Lan K., Wang H.S.-H., Lee T., de Assis C.A., Venditti R.A., Zhu Y., Yao Y. (2024). A Modeling Framework to Identify Environmentally Greener and Lower-Cost Pathways of Nanomaterials. Green Chem..

[241] Sun L., Hou C., Wei N., Tan Y., Liang Q., Feng J. (2023). pH/Cellulase Dual Environmentally Responsive Nano-Metal Organic Frameworks for Targeted Delivery of Pesticides and Improved Biosafety. Chem. Eng. J..

[242] Piroonpan T., Huajaikaew E., Kurantowicz N., Potiyaraj P., Pasanphan W. (2024). pH-Responsive Chitosan Nanoparticles for Controlled-Release Nitrogen Fertilizer: Template-Tampering Free Radical Graft Copolymerization under Energetic Radiation Study. Eur. Polym. J..

[243] Ma Y., Yu M., Wang Y., Pan S., Sun X., Zhao R., Sun Z., Gao R., Guo X., Xu Y. (2023). A pH/Cellulase Dual Stimuli-Responsive Cellulose-Coated Metal–Organic Framework for Eco-Friendly Fungicide Delivery. Chem. Eng. J..

[244] Chen X., Wang J., Hayat K., Zhang D., Zhou P. (2021). Small Structures with Big Impact: Multi-Walled Carbon Nanotubes Enhanced Remediation Efficiency in Hyperaccumulator *Solanum nigrum* L. under Cadmium and Arsenic Stress. Chemosphere.

[245] Chen Q., Cao X., Liu B., Nie X., Liang T., Suhr J., Ci L. (2021). Effects of Functional Carbon Nanodots on Water Hyacinth Response to Cd/Pb Stress: Implication for Phytoremediation. J. Environ. Manag..

[246] Kumar A.A., Kumar S.K.N., Fernandez R.E. (2021). Real Time Sensing of Soil Potassium Levels Using Zinc Oxide-Multiwall Carbon Nanotube-Based Sensors. IEEE Trans. NanoBioscience.

[247] Kumar M., Kaur N., Singh N. (2024). Colorimetric Nanozyme Sensor Array Based on Metal Nanoparticle-Decorated CNTs for Quantification of Pesticides in Real Water and Soil Samples. ACS Sustain. Chem. Eng..

[248] Loosli F., Yi Z., Wang J., Baalousha M. (2019). Improved Extraction Efficiency of Natural Nanomaterials in Soils to Facilitate Their Characterization Using a Multimethod Approach. Sci. Total Environ..

[249] Kah M., Tufenkji N., White J.C. (2019). Nano-Enabled Strategies to Enhance Crop Nutrition and Protection. Nat. Nanotechnol..

[250] Zhao Z., Wei H., Liu S., Xue Z. (2024). Estimation of agricultural soil surface roughness based on ultrasonic echo signal characteristics. Soil Tillage Res..

[251] European Commission (2023). Proposal for a Directive on Soil Monitoring and Resilience. COM(2023) 416 Final, Section 4.2.3 (“Innovative Remediation Technologies”). https://environment.ec.europa.eu/topics/soil-health/soil-health_en.

[252] Joint Research Centre Operational Framework for the EU Soil Mission Implementation. EUR 31517 EN, pp. 21–35, 2023. Publications Office of the EU. https://joint-research-centre.ec.europa.eu/eu-soil-observatory-euso_en.

[253] NANOREM Consortium (2016). Final Publishable Summary Report: Nanoremediation for Soil and Water. European Commission Grant Agreement No. 309517. https://cordis.europa.eu/project/id/309517.

[254] National Institute of Food and Agriculture (NIFA) (2017). Next-Generation Nanotechnology in Agriculture. USDA Project Portfolio: Grant No. A1511. https://www.nifa.usda.gov/data/data-gateway?path=fastlink1.txt&id=anon&pass=&search=(GC=A1511)%2520AND%2520(IY=2017)&format=WEBTITLESG.

[255] Brazilian Agricultural Research Corporation (Embrapa) (2022). Fertgel Hydrogel for Nutrient and Water Management in Semi-Arid Tropics (Technical Bulletin No. 245). https://www.embrapa.br/agroenergia.

